# Adult Onset Global Loss of the *Fto* Gene Alters Body Composition and Metabolism in the Mouse

**DOI:** 10.1371/journal.pgen.1003166

**Published:** 2013-01-03

**Authors:** Fiona McMurray, Chris D. Church, Rachel Larder, George Nicholson, Sara Wells, Lydia Teboul, Y. C. Loraine Tung, Debra Rimmington, Fatima Bosch, Veronica Jimenez, Giles S. H. Yeo, Stephen O'Rahilly, Frances M. Ashcroft, Anthony P. Coll, Roger D. Cox

**Affiliations:** 1 MRC Harwell, Harwell Science and Innovation Campus, Harwell, United Kingdom; 2 University of Cambridge Metabolic Research Laboratories and NIHR Cambridge Biomedical Research Centre, Institute of Metabolic Science, Addenbrooke's Hospital, Cambridge, United Kingdom; 3 Department of Statistics, University of Oxford, Oxford, United Kingdom; 4 Center of Animal Biotechnology and Gene Therapy and Department of Biochemistry and Molecular Biology, School of Veterinary Medicine, Universitat Autònoma de Barcelona, Bellaterra, Spain; 5 Centro de Investigación Biomédica en Red de Diabetes y Enfermedades Metabólicas Asociadas (CIBERDEM), Barcelona, Spain; 6 Henry Wellcome Centre for Gene Function, Department of Physiology, Anatomy, and Genetics, University of Oxford, Oxford, United Kingdom; University of Cincinnati, United States of America

## Abstract

The strongest BMI–associated GWAS locus in humans is the *FTO* gene. Rodent studies demonstrate a role for FTO in energy homeostasis and body composition. The phenotypes observed in loss of expression studies are complex with perinatal lethality, stunted growth from weaning, and significant alterations in body composition. Thus understanding how and where *Fto* regulates food intake, energy expenditure, and body composition is a challenge. To address this we generated a series of mice with distinct temporal and spatial loss of *Fto* expression. Global germline loss of *Fto* resulted in high perinatal lethality and a reduction in body length, fat mass, and lean mass. When ratio corrected for lean mass, mice had a significant increase in energy expenditure, but more appropriate multiple linear regression normalisation showed no difference in energy expenditure. Global deletion of *Fto* after the *in utero* and perinatal period, at 6 weeks of age, removed the high lethality of germline loss. However, there was a reduction in weight by 9 weeks, primarily as loss of lean mass. Over the subsequent 10 weeks, weight converged, driven by an increase in fat mass. There was a switch to a lower RER with no overall change in food intake or energy expenditure. To test if the phenotype can be explained by loss of *Fto* in the mediobasal hypothalamus, we sterotactically injected adeno-associated viral vectors encoding Cre recombinase to cause regional deletion. We observed a small reduction in food intake and weight gain with no effect on energy expenditure or body composition. Thus, although hypothalamic *Fto* can impact feeding, the effect of loss of *Fto* on body composition is brought about by its actions at sites elsewhere. Our data suggest that *Fto* may have a critical role in the control of lean mass, independent of its effect on food intake.

## Author Summary

The fat mass and obesity (FTO) gene has one of the strongest links with body mass index (BMI) in the human population. One in six people have the “risk” alteration and weigh 3 kg more than those with the unaltered gene, but it is not understood how this gene influences BMI and obesity. We set out to understand how and where in the body FTO affects food intake, energy expenditure, and body composition using a mouse model that can be manipulated to lack FTO at particular times and/or places. Removing FTO everywhere from conception had a dramatic effect on body composition and resulted in stunted growth and some lethality. Removing FTO everywhere but only in adult animals resulted in better viability and normal growth but, surprisingly, reduced lean mass and increased fat mass with a change in the type of metabolic fuel being used. Finally, we removed FTO from the hypothalamus of adult animals, an important brain region involved in energy metabolism. These animals showed a mild reduction in food intake and weight gain. Our experiments show that FTO has an important role in body composition and that other brain areas outside of the hypothalamus are also important in determining its effects.

## Introduction

The fat mass and obesity-associated (*FTO*) gene is an AlkB-like, Fe(II)- and 2-oxoglutarate–dependent nucleic acid demethylase that has been shown to demethylate 3-methylthymine and 3-methyluracil in single-stranded DNA and RNA, respectively [1]–[2]. More recently FTO has been demonstrated to demethylate N6-methyladenosine in nuclear RNA [3].

Several single nucleotide polymorphisms (SNPs) in the first intron of the *FTO* gene are associated with obesity and type 2 diabetes [4]–[7] (reviewed [8]–[9]). Approximately 16% of individuals of European descent are homozygous for the at-risk (A) allele, and have a ∼1.67-fold increased risk of obesity, weighing on average ∼3 kg more than those with the T allele (rs9939609) [5]. The effect of *FTO* on BMI is observed by 7 years of age [5]. A study looking at BMI from early infancy to 13 years old has revealed a more complex pattern with the rs9939609 A allele associated with lower BMI before the age of 2.5 years and then a positive (increased BMI) association after the age of 5.5 years [10]. This appears to be due to an earlier adiposity rebound (normal BMI peaks in the first year and then there is decline in adiposity up to about 5 years of age after which it then increases (rebounds) again) in individuals with the A allele [10]–[11]. Life course studies with rs9939609 show BMI association strengthening during childhood and adolescence up to a peak around 20 years of age and then declining [12].

A homozygous R316Q enzyme inactivating mutation resulted in a broad spectrum of clinical manifestations including severe intrauterine growth retardation and death before the age of three [13]. The early developmental effects of FTO mutations and the profile of BMI effects of *FTO* risk alleles in children and young people suggest that FTO may be important in development *in utero* and early life. However, it is noteworthy that heterozygous loss-of-enzymatic-function mutations can occur in both lean and obese individuals [14].

Many human population studies have shown that at least some FTO effects may be mediated through increased food intake (in some cases of high fat or more palatable food) and effects on hunger and satiety responses [15]–[22]. However, a high fat diet and inactivity may instead be a modifying factor interacting with *FTO* genotype [23]–[25].

In the mouse, two constitutive knockout alleles have been reported (Fto^tm1Urt^ and Fto^tm1.1Pzg^), both of which cause postnatal growth retardation and high postnatal mortality rates [26]–[27]. In one of these studies there was significant reduction in adipose tissue and lean body mass that was attributed to increased energy expenditure despite relative hyperphagia [26]. However, this study used division by lean body mass to account for the changed body composition, rather than multiple linear regression of body composition parameters using ANCOVA as is commonly used in human studies [28]–[30]. This led to much discussion in the literature of energy expenditure normalization methods. In the second study, body composition was relatively normal although there was lower body weight, lower bone mineral density, shorter body length and relative hyperphagia [27]. Energy expenditure was increased, both when determined by ratio analysis against lean mass as well as ANCOVA. Deletion of *Fto* in neurons (using Nestin-Cre) caused a similar phenotype, demonstrating a key neuronal role for FTO in postnatal growth [27]. A third loss-of-function mouse model comprising a dominant missense mutation in the C-terminal of the mouse *Fto* gene resulted in reduced weight and fat mass and increased energy expenditure without the increased perinatal death and growth retardation [31]. In contrast, a mouse model that globally overexpressed FTO showed increased food intake, body weight and fat mass [32].

These animal models clearly indicate a role for *Fto* in energy metabolism, but many questions remain unresolved, including whether there is any effect on energy expenditure, what the role of *Fto* is in early life and in the adult, which tissues are involved, which regions of the brain contribute to the phenotype and what is the cellular role of FTO.

In our studies we sought to resolve the question of energy expenditure in global germline knockout mice. We found no evidence for increased energy expenditure in this or our other two models analysed using ANCOVA. We used our conditional mouse knockout allele with a tamoxifen inducible Cre recombinase to circumvent the perinatal lethality and growth retardation observed in global germline knockouts in order to investigate the role of FTO in adult animals. In adult onset global knockout mice we observed reduced body weight with later weight convergence and - surprisingly - reduced lean mass and increased fat mass. The latter explains the later weight convergence. We further asked whether these effects were due to loss of FTO in mediobasal hypothalamic regions of the brain and found that these mice were only mildly affected, suggesting a role for FTO in brain regions other than the hypothalamus.

## Results

In order to investigate the function of the *Fto* gene in the mouse we constructed mice carrying a LoxP-flanked (floxed) exon 3 frameshift allele (Figure S1A). These mice were used to generate animals in which *Fto* was knocked out in all tissues either prior to birth or in adult life. Male mice were used in global germline knockout and adult onset knockout experiments and female mice in adult onset hypothalamic knockout experiments.

### Global Germline Knockout Results in Perinatal Lethality and Reduced Size

*Fto* exon 3 and the PGK-neomycin selection cassette were excised by Cre recombinase, under the control of the β-actin promoter, to generate a constitutive global germline *Fto* knockout (germline KO) allele (Figure S1A). Heterozygous germline KO mice were intercrossed to generate heterozygous (*Fto* ^+/−^), homozygous (*Fto* ^−/−^) and wild-type (*Fto* ^+/+^; WT) littermate controls. Deletion of *Fto* exon 3 was shown by RT-PCR (data not shown) and loss of FTO protein expression was confirmed by immunoblotting (Figure S1B).

**Figure 1:**
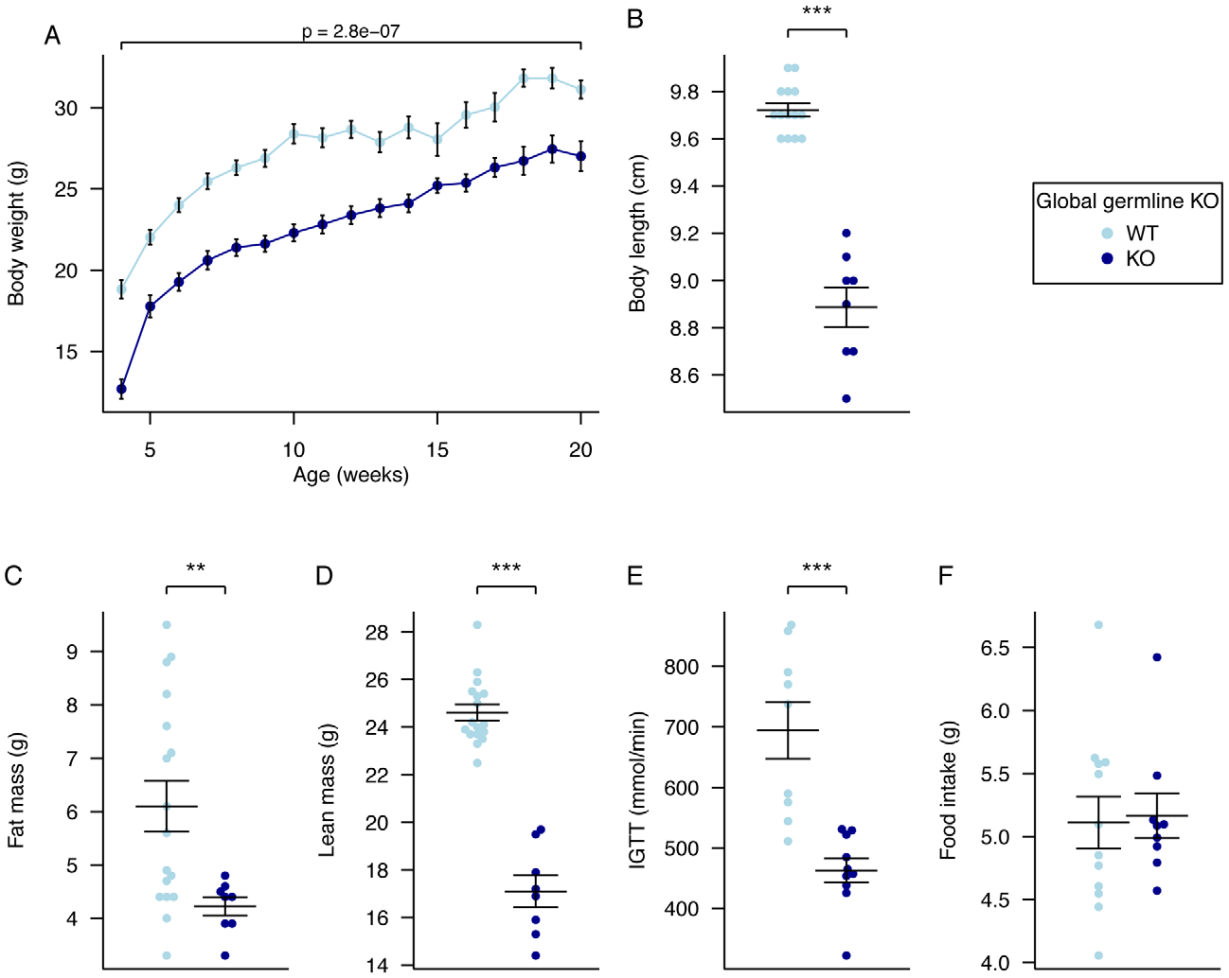
Body weight, body composition, and food intake in male global germline *Fto* Knockout mice. A, Male body weight WT *Fto* ^+/+^, (n = 10), *Fto* ^−/−^ (n = 10), B, Body length WT *Fto* ^+/+^, (n = 14), *Fto* ^−/−^ (n = 8), C, Fat mass in males at 20 weeks of age, WT *Fto* ^+/+^, (n = 17), *Fto* ^−/−^ (n = 8), D, Lean mass in males at 20 weeks of age, WT *Fto* ^+/+^, (n = 17), *Fto* ^−/−^ (n = 8), E, Intraperitoneal glucose tolerance test (IPGTT) area under the curve (AUC) in males at 16 weeks of age. WT *Fto* ^+/+^, (n = 9), *Fto* ^−/−^ (n = 10), F, Food intake in male mice at 10 weeks of age during a 24-hour period in metabolic cages. WT *Fto* ^+/+^, (n = 12), *Fto* ^−/−^ (n = 9). Data are expressed as mean ± SE and in B–F individual data points are shown. Time-course data were analysed using the repeated-measures ANOVA model (see Materials and Methods) (A). The other comparison p-values (B–F) correspond to a Welch *t*-test of the null hypothesis of no difference between genotypic groups. *P<0.05, **P<0.01, ***P<0.001.

Homozygous germline *Fto* ^−/−^ mice show perinatal lethality with only 45% surviving to weaning (data not shown). Body weight was measured weekly from 4 to 20 weeks of age. Male *Fto* ^−/−^ mice weighed significantly less than WT littermates at all ages being approximately 13% lighter at 20 weeks (Figure 1A, Table S1). Additionally, *Fto* ^−/−^ mice displayed growth restriction (anus to nose length approximately 0.7 cm shorter) when compared to WT littermates (Figure 1B). Body composition, as determined by dual energy X-ray absorptiometry (DEXA), showed a reduction in fat and lean mass in KO males when compared to WT littermates (Figure 1C, 1D). When corrected for body weight, the percentage reduction in fat mass was not significant but lean mass was significantly reduced (by 11.5% in males; p = 2.50E-05 by Welch t-test of raw data, p = 0.02 after multiple linear regression normalisation for body weight Table S2). Measurement of glucose tolerance in a 30-minute intraperitoneal glucose tolerance test (IPGTT) showed some evidence of improved tolerance in male homozygotes (Figure 1E).

### Global Germline Knockout Results in Altered RER and Unchanged Energy Expenditure

Absolute food intake (g/day) was not significantly different between homozygous *Fto* ^−/−^ mice and WT littermates (Figure 1F); however, relative food intake was increased when normalised for the significantly reduced body weight (data not shown).

**Figure 2:**
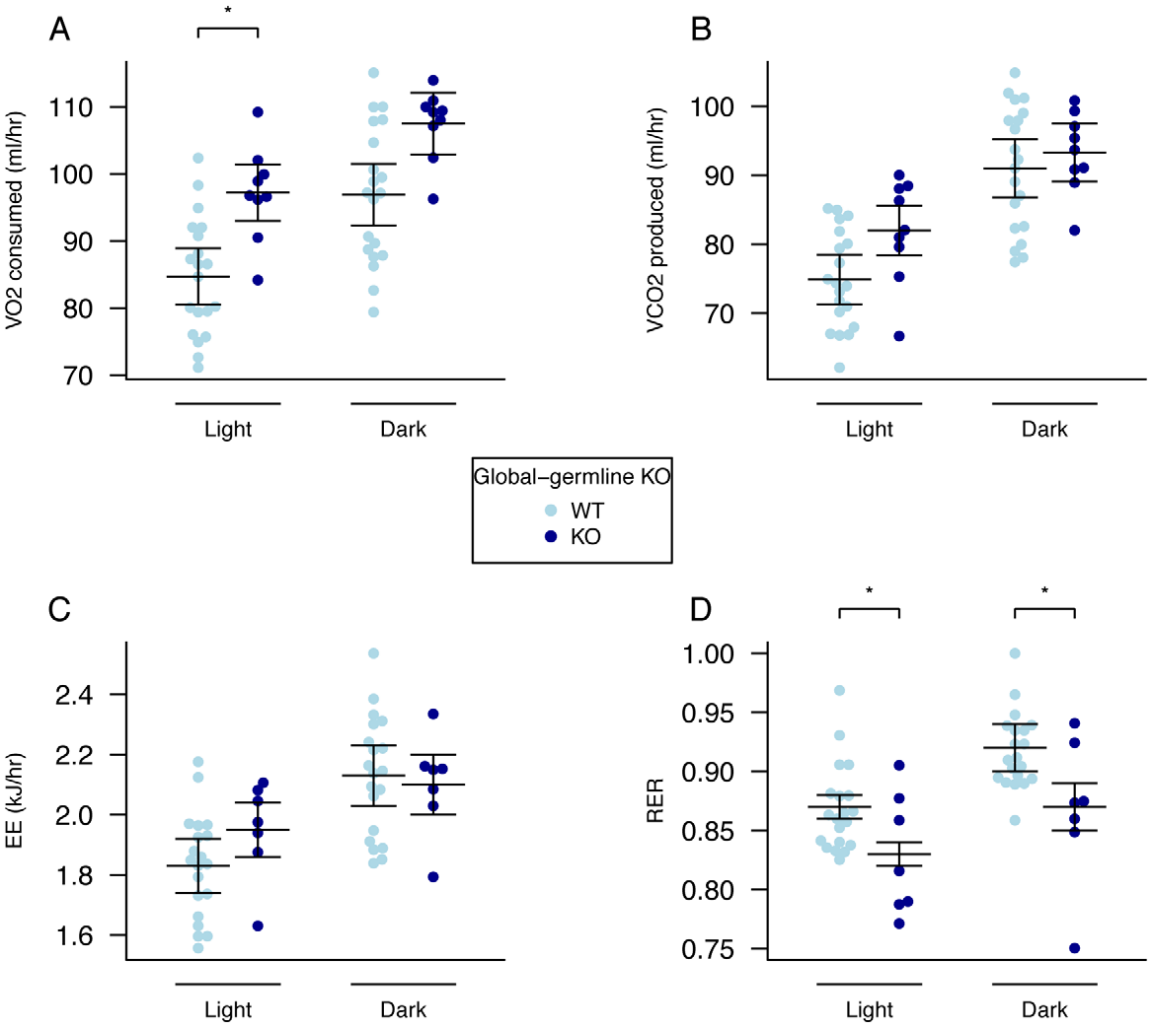
Energy expenditure and metabolism in male global germline *Fto* KO mice. A, VO_2_ consumed, B, VCO_2_ produced, and C, energy expenditure (EE), adjusted for variation in lean mass using multiple linear regression (ANCOVA). D, respiratory exchange ratio (RER). Measurements were made in male mice during the light and dark phases at 18-weeks of age. *Fto* ^+/+^ (n = 20), *Fto* ^−/−^ (n = 9). Data are expressed as mean ± SE and individual data points are shown. For details of the lean-mass adjustment made in panels A–C, see Materials and Methods, *P<0.05, **P<0.01, ***P<0.001.

Oxygen consumption, corrected for lean body mass, was increased in homozygous male *Fto* ^−/−^ mice in both the light and dark phases, but the difference was statistically significant only in the light phase (Figure 2A, Table S3). Carbon dioxide output and energy expenditure, both lean mass corrected, were not significantly different (Figure 2B, 2C, Table S3). However, the RER was significantly reduced in both light and dark phases (Figure 2D, Table S3).

**Figure 3:**
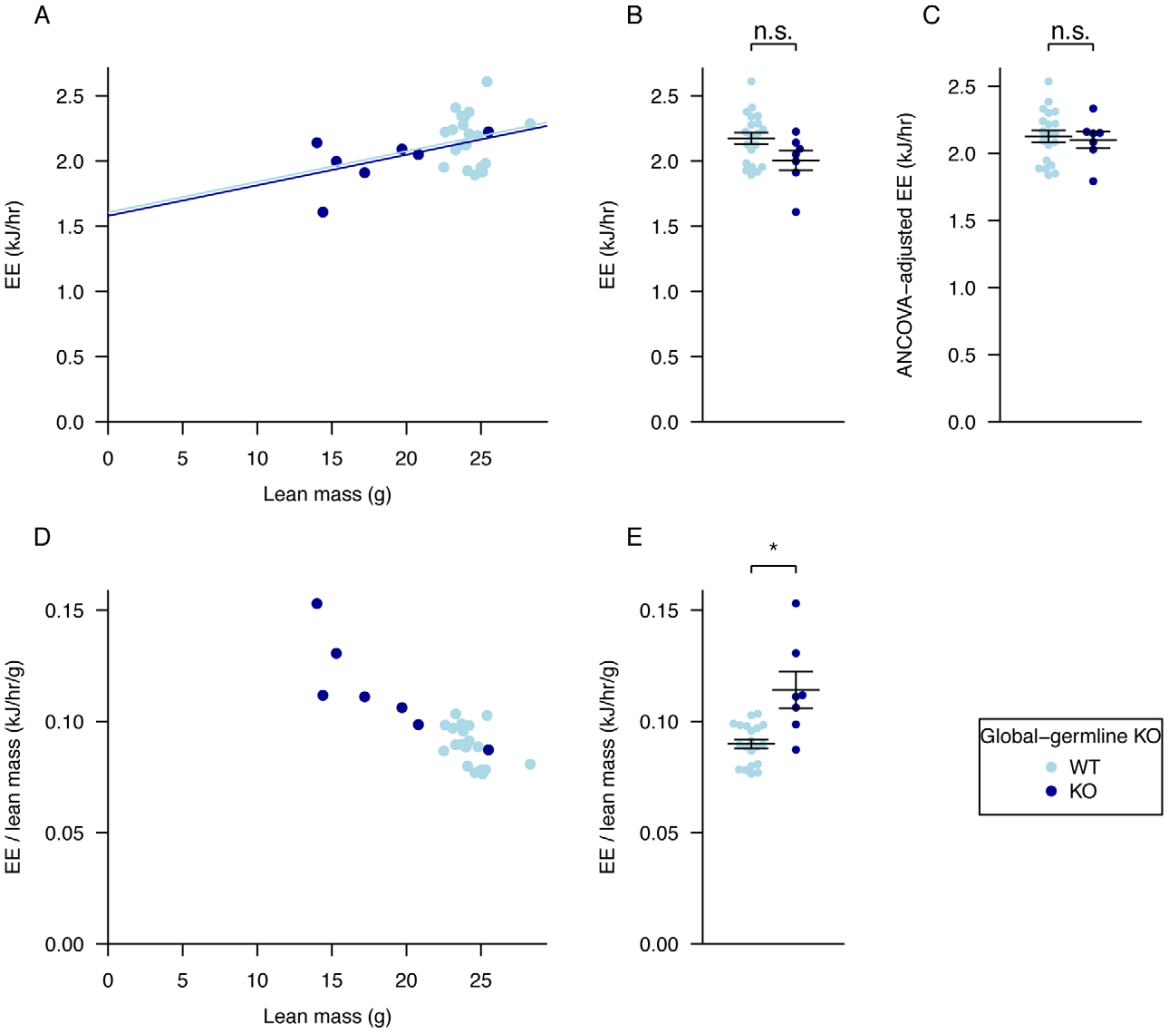
Adjustment of energy expenditure for lean mass, regression analysis compared to ratio adjustment. A, regression of energy expenditure against lean mass, B, average energy expenditure, C, energy expenditure values adjusted for lean mass using ANCOVA, D, values for energy expenditure divided by lean mass and plotted against lean mass to show the effect of ratio adjustment, E, average energy expenditure divided by lean mass. Data as in Figure 2 for dark phase. The p-values in B,E were calculated using a Welch *t*-test of the null hypothesis of no difference between genotypic groups. For lean mass adjustment in C see Materials and Methods. *P<0.05.

There has been much discussion in the literature of the correct way to normalise energy expenditure data for FTO KO mice [28]. Because our mutant mice are substantially lighter than their WT littermates we compared linear regression and ratio normalisation methods (Figure 3A–3E). To illustrate the differences between these two methods, we plotted energy expenditure against lean mass (Figure 3A). When energy expenditure data are not corrected (Figure 3B), WT mice exhibit higher mean energy expenditure than KO mice (although the difference is not statistically significant with our sample size). However, as Figure 3A shows, WT mice have higher lean mass, and lean mass positively correlates with energy expenditure. Consequently the difference in energy expenditure disappears upon regression adjustment for lean mass (Figure 3C). The difference in regression-adjusted energy expenditure in Figure 3C is the vertical distance between the two lines in Figure 3A (see Materials and Methods for details). The ratio-normalization approach (Figure 3D, 3E) over-corrects for the effects of lean mass, leading to artificial inflation of ratio-adjusted energy expenditure in KO mice relative to WT mice. This problem is now widely recognized and it is accepted that regression based adjustment is the most appropriate way to account for differences in body weight or composition, as has been the case in human studies for many years [29]–[30]. We conclude that in our global germline knockout there is no detectable difference in energy expenditure rate between knockout and WT littermates.

Our global *Fto* germline knockout exhibits many similarities to the two other published *Fto* knockouts [26]–[27]. However, as all these models show considerable perinatal lethality and growth restriction it is difficult to determine the precise role of FTO in body composition and metabolism. In order to circumvent early postnatal and developmental effects that lead to lethality and growth restriction as a result of FTO loss, we therefore inactivated *Fto* in adult mice.

### The Severe Lethality Seen with Germline *Fto* Loss Is Not Seen in Adult Onset Loss

Mice carrying the conditional floxed *Fto* allele were crossed with mice carrying a tamoxifen-inducible ubiquitin-Cre recombinase and the resulting heterozygous mice carrying Cre recombinase intercrossed to generate homozygous floxed *Fto* mice and homozygous mice carrying both floxed *Fto* and Cre recombinase. Mice carrying Cre recombinase alone were bred from the heterozygote population by crossing with WT mice. All mice were on a congenic C57BL/6J background. Mice of all genotypes were treated with tamoxifen or vehicle at 6 weeks of age (around the time of sexual maturity) by oral gavage daily for five days. This led to global (as ubiquitin is expressed in most, if not all, tissues) deletion of *Fto* in mice carrying both floxed *Fto* and Cre recombinase: we refer to these as adult onset KO mice. Inactivation of the *Fto* gene and loss of FTO protein was demonstrated by immunoblotting for FTO (Figure S2).

There were 3 unexplained deaths out of 25 adult onset KO mice (12%) over the 14 weeks after tamoxifen treatment, and one death in the vehicle control group (n = 11, 9%). This indicates substantially improved viability compared with germline KO mice (45% lethality). Whether the 3 deaths in adult KO onset mice are related to *Fto* deletion or are chance events is unknown.

Repeated measures analysis comparing post-treatment body weight data from vehicle-treated mice carrying both Cre and floxed *Fto* alleles (Vehicle) and tamoxifen-treated Cre mice (Tamoxifen) did not show significant differences in weight (*p* = 0.89). We therefore present below only data on male mice carrying both the floxed *Fto* allele and Cre recombinase, which have either been treated with tamoxifen (Adult onset KO) or with the corn oil and 2% ethanol vehicle (control).

### Adult Onset KO Mice Have Reduced Body Weight and No Growth Retardation

**Figure 4:**
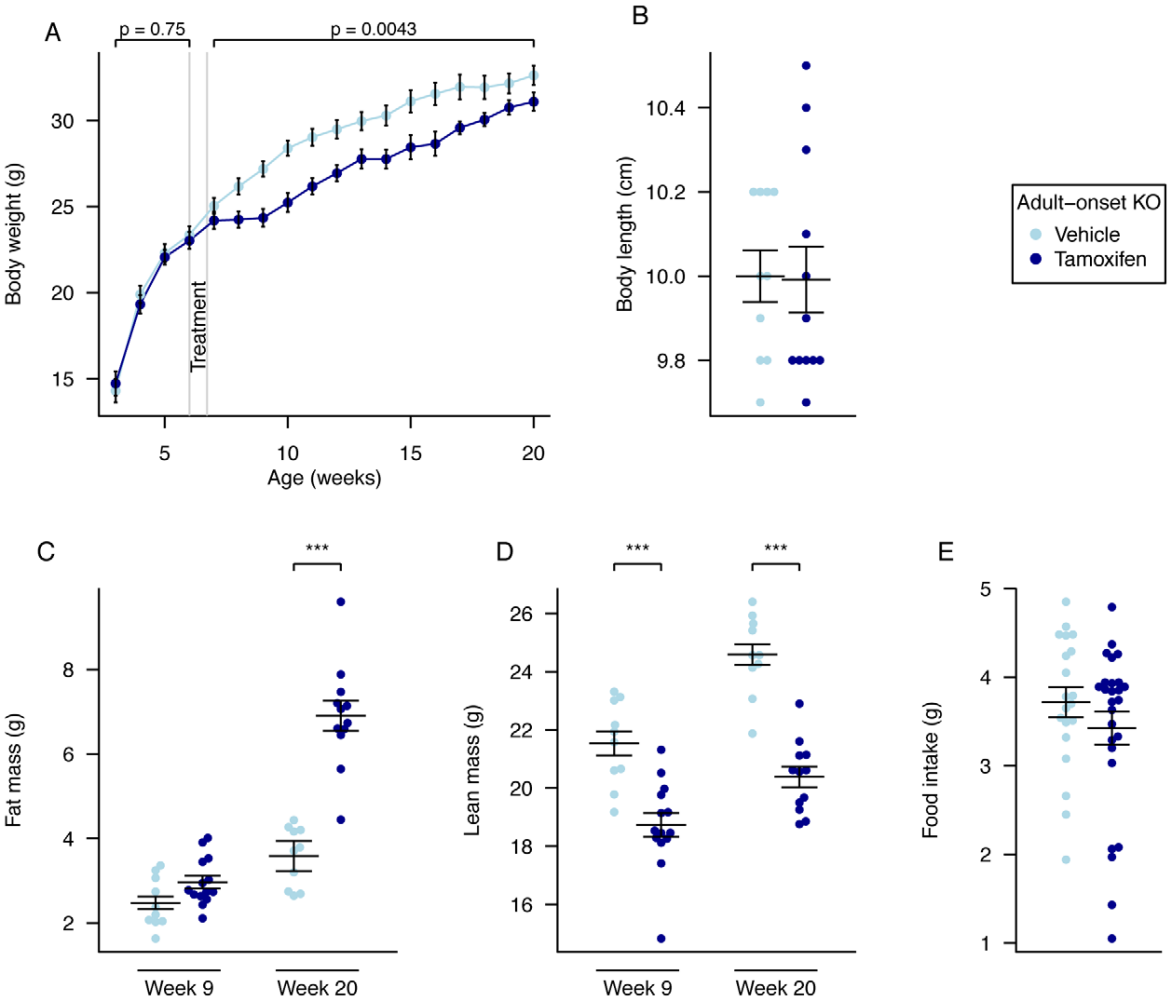
Body weight, body composition, and food intake of male global adult onset KO mice. A, Weekly body weight of adult onset KO (n = 15) and vehicle-treated control mice (n = 10), B, Nose-to-anus body length of control (n = 10) and adult onset KO mice (n = 12) at 20 weeks of age, C, Fat mass was increased in adult onset KO mice (n = 14) compared to controls (n = 10) in 9-week old mice (P = 0.05) and further increased in adult onset KO mice (n = 12) compared to controls (n = 10) at 20 weeks, D, Lean mass was reduced in adult onset KO mice (n = 14 and n = 12) compared to controls (n = 10 and n = 10) at both 9 weeks and 20 weeks of age, E, Food intake at 10 weeks of age during a 24-hour period in metabolic cages, control (n = 10) and adult onset KO mice (n = 14). Data are expressed as mean ± SE. In B–E, individual data points are shown. Time-course data were analysed using the repeated-measures ANOVA model, see Materials and Methods (A). The other comparison p-values (B–E), correspond to a Welch *t*-test of the null hypothesis of no difference between genotypic groups. *P<0.05, **P<0.01, ***P<0.001.

Mice were weighed weekly from 3 weeks of age. In the weeks prior to treatment (weeks 3 to 6) there was no statistical difference between groups (Figure 4A; Table S4). Mice were treated with tamoxifen or vehicle at 6 weeks of age. During weeks 7 to 9, the average rate of weight gain of adult onset KO mice was slower than that of control mice, leading to a lower mean weight of KO mice that persisted over the next 12 weeks (Figure 4A). Repeated-measures ANOVA indicated statistically significant inter-group differences, with reduced KO body mass relative to controls (p = 0.0043). Weekly inter-group differences were statistically significant throughout weeks 8 to 18 (2 to 14 weeks after treatment started (Time-by-time ANOVA p<0.05; Table S4).

There was no change in body length when measured in sacrificed animals at 20 weeks of age (Figure 4B).

### Adult Onset KO Mice Have Increased Fat and Decreased Lean Mass

Quantitative NMR was used to determine lean and fat mass weights at 9 and 20 weeks of age (3 and 14 weeks after treatment started, Figure 4C, 4D). At 9 weeks of age there was a significant reduction in lean mass (p = 0.00017), a fall in lean mass as a proportion of body weight (p = 8.9E-05), a trend to increased fat mass (p = 0.050), and a significant increase in fat mass as a proportion of body weight (p = 0.0047), in KO mice compared to controls (Figure 4C, 4D and Table S2). Using multiple linear regression to better take account of the body weight differences, lean mass was significantly decreased (p = 0.0011) and fat mass was increased (p = 0.024; Table S2). These differences were considerably larger at 20 weeks of age (Body weight adjusted ANCOVA: lean mass p = 2.0E-09, fat mass p = 5.9E-10, Figure 4C, 4D).

### Adult Onset Global KO Mice Do Not Have Increased Energy Expenditure but Do Show Altered Metabolism

To further understand these weight and body composition differences we measured food intake and energy expenditure.

**Figure 5:**
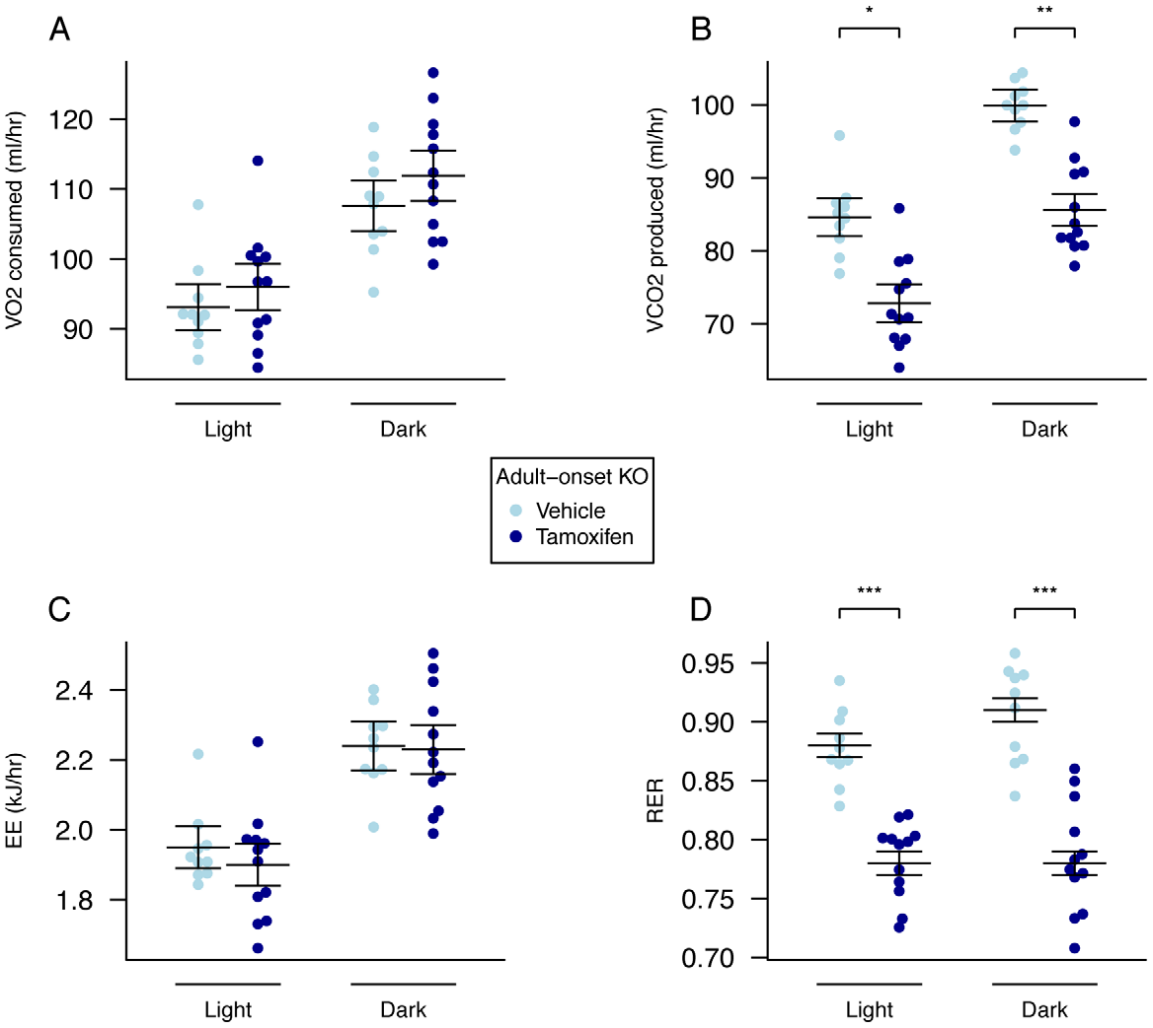
Energy expenditure and metabolism of male global adult onset KO mice. A, VO_2_ consumed, B, VCO_2_ produced, and C, energy expenditure (EE), adjusted for variation in lean mass using multiple linear regression (ANCOVA). D, respiratory exchange ratio (RER). Adult onset KO mice (n = 12) control mice (n = 10). Measurements were made over a 22-hour period during the dark and light phases in 18-week old mice. Data are expressed as mean ± SE and individual data points are shown. For details of the lean-mass adjustment made in panels A,B,C, see Materials and Methods, *P<0.05, **P<0.01, ***P<0.001.

There were no significant differences in 24-hour food intake measurements in metabolic cages at either 10 (Figure 4E) or 19 weeks (data not shown) of age. Adult onset KO mice were subjected to 24-hour indirect calorimetry at 18 weeks of age and showed unchanged oxygen consumption and decreased carbon dioxide output (when adjusted for lean body mass Figure 5A and 5B, Table S3). Energy expenditure adjusted for lean mass was not significantly different during either light or dark periods (Figure 5C, Table S3). The RER of adult KO mice was significantly reduced during both periods and overall (Figure 5D, Table S3).

### Adult Onset Hypothalamic *Fto* Loss Has a Small, but Significant, Effect on Body Weight due to Decreased Food Intake

As FTO has been proposed to affect hypothalamic metabolism control pathways, we next tested whether loss of FTO in the hypothalamus recapitulated some or all of the adult KO phenotype. We used adeno-associated viral (AAV) vector delivery technology of Cre recombinase to delete the *Fto* gene specifically within the mediobasal hypothalamus of 9–11 week old female *Fto*-floxed mice (Figure S3A–S3H). AAV vectors of serotype 7 were generated to produce either Cre recombinase (AAV-Cre) or GFP (AAV-GFP), as previously described [33]. Control experiments were performed to verify the effectiveness of the AAV-Cre vectors in removing *Fto* expression *in vivo* (Figure S3A, S3B and Figure S4A, S4B).

**Figure 6:**
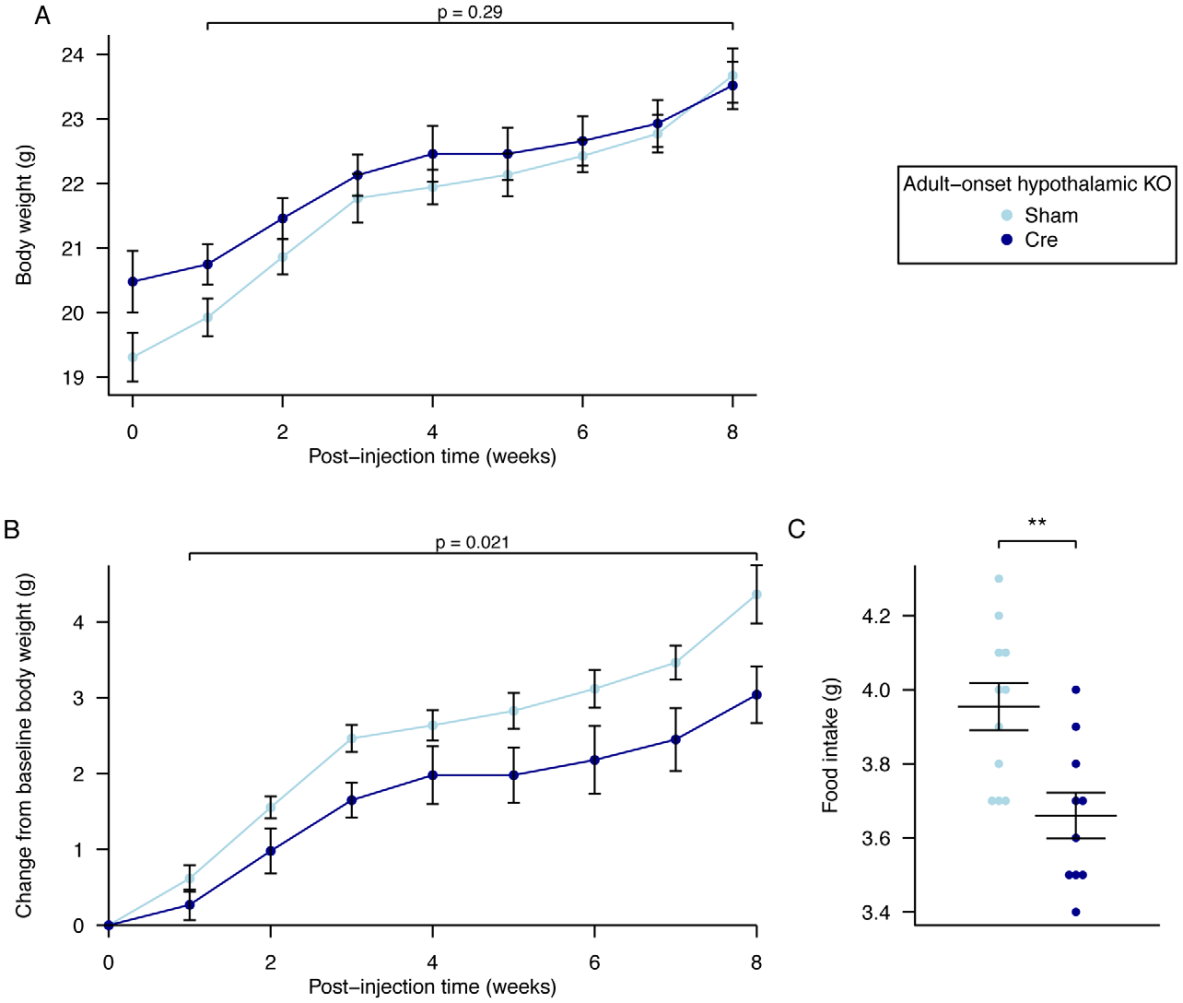
Body weight, weight gain, and food intake in female adult hypothalamic KO mice. A, Body weight post injection, B, change in body weight after injection of either an AAV-GFP (Sham) or AAV-Cre (Cre) vectors, C, Average daily food intake between days 14 and 23 post surgery. AAV Cre treated (n = 10) and Sham control (n = 11). Data are expressed as mean ± SE and in C individual data points are shown. Time-course data were analysed using the repeated-measures ANOVA model, see Materials and Methods (A,B). The average food intake p-value (C) corresponds to a Welch *t*-test of the null hypothesis of no difference between genotypic groups, **P<0.01.

**Figure 7:**
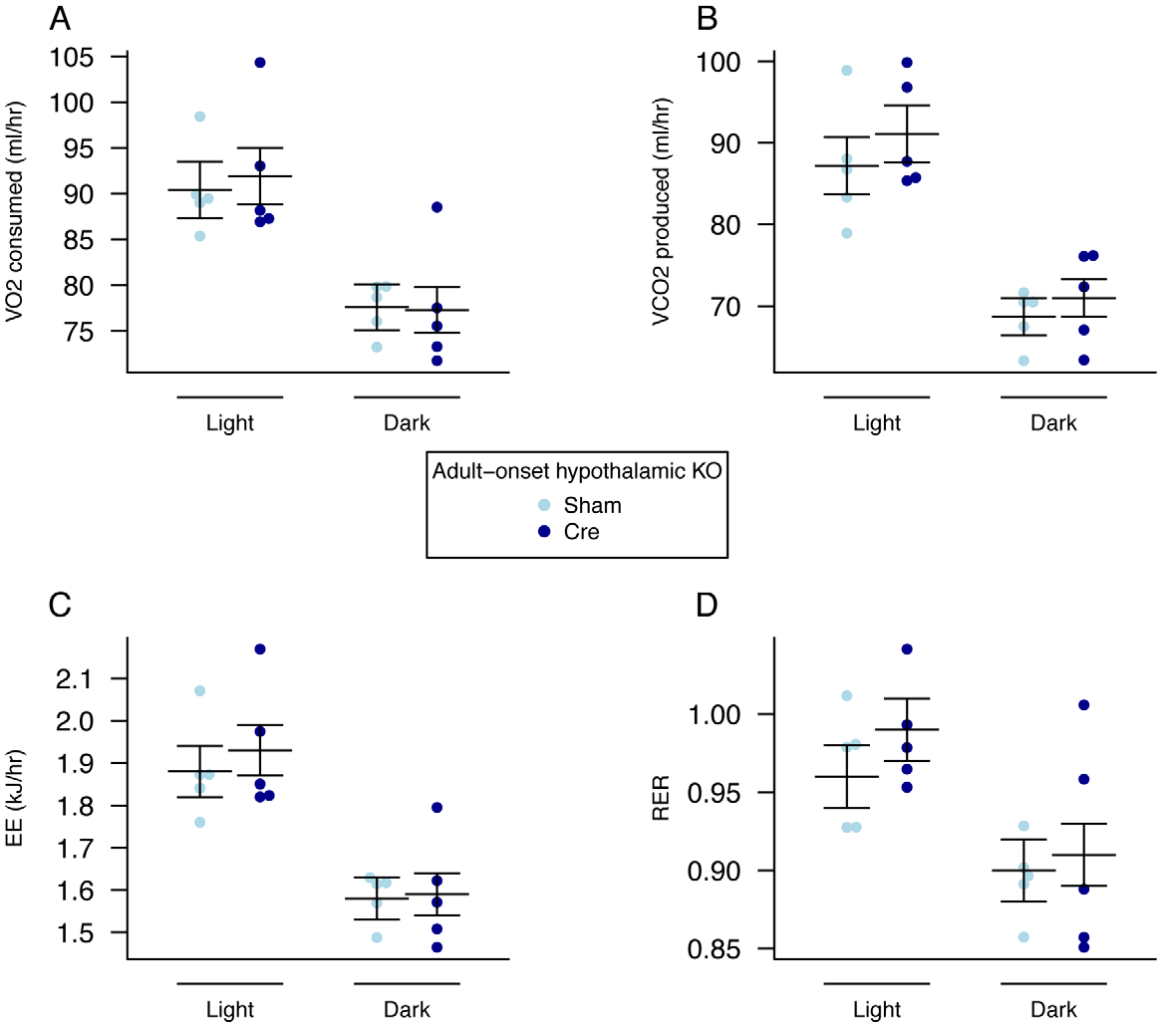
Energy expenditure and metabolism in female adult hypothalamic KO mice. A, VO_2_ consumed, B, VCO_2_ produced, and C, energy expenditure (EE). adjusted for variation in lean mass using multiple linear regression (ANCOVA). D, respiratory exchange ratio (RER). Female adult AAV-Cre treated (n = 5) and sham control (n = 5) mice. Measurements were made, 5 weeks after treatment, over a 48-hour period during the dark (lights out) and light (lights on) phases. Data are expressed as mean ± SE and individual data points are shown. For details of the lean-mass adjustment made in panels A,B,C, see Materials and Methods. No significant differences were found.

Over an 8-week period, AAV-Cre injected mice did not show a significant difference in weight (Figure 6A, Table S5) although they gained significantly less body weight than AAV-GFP injected controls (Figure 6B, Table S6). No clearly demonstrable body composition difference was seen between the 2 groups on DEXA analysis (data not shown). Measurement of daily food intake between days 14–23 post injection revealed that the decreased weight gain was likely due to a significant reduction in food intake in animals lacking expression of *Fto* within the hypothalamus (Figure 6C). There was no demonstrable difference in energy expenditure between sham and Cre-treated mice (Figure 7A–7D, Figure S5, Table S3). Similar effects were seen in male *Fto*-floxed mice (data not shown).

## Discussion

The phenotype of our mouse carrying a constitutive global KO of *Fto* is very similar to the two previously published models [26]–[27]. They showed marked perinatal mortality and a clear divergence of body weight from controls early in the perinatal period [26]–[27]. This may reflect changes that occurred *in utero* and/or a requirement for FTO from birth.

There has been some discussion about the most appropriate way to correct energy expenditure for differences in body weight and composition in both *Fto* KO mice and mouse models in general [28], [30], [34]–[35]. Using multiple linear regression to control for variation in lean mass, we found no significant difference in energy expenditure between the KO and littermate controls in both light and dark phases. This is in contrast to the study by Gao *et al.* [27] who reported an increased energy expenditure tested by ANCOVA. Using the ratio method, in which data were analysed by dividing energy expenditure by lean mass, our results show increased energy expenditure in line with both previous studies. However, ratio analyses are confounded by the very different lean masses of the knockout and littermate control groups [26]–[27]. ANCOVA analysis, which is more correct, reveals that our germline KO mice show no differences in energy expenditure. The different result obtained by Gao et al. [27], is likely to be explained by differences in body composition in their mouse model.

To avoid the high perinatal lethality of germline KO mice, and to address the role of *FTO* gene in adult mice, we generated an inducible global KO mouse and inactivated the *Fto* gene at 6 weeks of age. These mice showed much improved viability compared to mice with a germline KO of *Fto*. However, some increased mortality with respect to controls remained. The cause of this remains unclear and prior to their deaths the mice were not reported to have been unwell at daily check. Given the phenotype of children with enzymatic null mutations, one may speculate that the demise of the 3 mice in question could have been linked to a cardiac phenomenon but this is far from certain.

Adult onset KO mice showed a reduction in body weight in the weeks immediately following gene deletion, but convergence of body weight towards the last 4 weeks of the study (17–20 weeks of age). The lower weight is consistent with data from both our own and published germline global KO mice. Interestingly, in a previous study, female germline global *Fto* KO mice also showed convergence of body weight with controls by about 13 weeks of age [27]. Energy expenditure in 18-week old mice, when adjusted for lean mass variation using multiple linear regression, was not significantly different between the two groups. We also did not detect any significant differences in 24-hour food intake measurements. It would be interesting to know if the differences in body weight are due to changes in food intake or expenditure - small differences in these parameters (too small to detect in our experiments) could have a cumulative effect over a long time period. Other possible explanations for the reduced body weight include changes in the efficiency with which energy is extracted from food and changes in metabolism.

The significantly reduced lean mass at 9 and 20 weeks of age in adult onset KO mice (compared to controls) is consistent with data from the germline KO mice. This derives from a lower rate of gain of lean mass rather than lean mass wasting. The most striking observation in the adult onset KO mice was the marked gain in fat mass (both fat weight and fat expressed as a percentage of body weight increased). By 20 weeks of age the fat weight in the adult onset KO was almost double (1.92×) that of controls. This contrasts with all three constitutive KO models, which showed a reduction in fat mass. Given the lower rate of gain of lean mass it would be interesting to look at the effect of *Fto* loss in older animals in which growth had plateaued to examine how the loss of *Fto* affects established body composition.

When body weight was taken into account, neither our germline global KO mouse nor that of Gao *et al.* [27] showed any difference in the percentage of fat mass in males. However, in the latter study, female mice showed a significant increase in percentage fat, consistent with our adult onset KO mice. The gain in fat mass in adult onset KO mice was more significant at 20 weeks of age when body weight differences were smaller. Thus, the lower weight of adult onset KO mice appears to be due, at least in part, to a lower lean body mass. Further, due to a redistribution of lean mass and fat mass, combined with an increase in fat mass, body weight was no longer significantly different in adult onset KO and control mice 20 weeks after deletion of *Fto*. At 20 weeks of age, adult onset KO mice weigh, on average, 1.5 g less than controls: around 0.9 g of this difference is not explained by the observed changes in lean and fat mass, and may possibly be attributable to changes in skeletal growth. Consistent with this the germline knockout male mice showed reduced bone mineral content and density (unpublished observations).

Our data clearly indicate that FTO affects body composition. Early global loss of FTO is more severe perhaps due to critical events during the suckling and weaning periods, and a dietary response that leads to growth deficits. Adult onset loss of FTO results in increased fat mass at the expense of lean body mass and reduced weight without overt linear growth restriction.

We observed altered carbon dioxide output in adult onset KO mice. This resulted in a large reduction in RER. That this occurred with a reduction in lean body mass may be in keeping with increased protein utilisation at the expense of carbohydrate. The observed metabolic switch is consistent with the reduction in lean body mass. How loss of FTO causes this metabolic switch is an important question for future research. Recently, Cheung et al (2012) have shown that FTO expression is down-regulated *in vitro* by essential amino-acid deprivation [36]. Given the effects on lean mass and RER in adult onset KO mice this may be an interesting avenue for future investigation.

The association of *FTO* SNPs with food intake in the human population suggests at-risk alleles are associated with increased food intake [15]–[22]. Additionally, the increased food intake observed in constitutive KO mice (when account is taken of the reduced body mass) and in a mouse overexpressing *Fto* focus attention on the hypothalamic centres of the brain involved in controlling food intake [26], [27], [32]. However, our deletion of *Fto* in the hypothalamus using AAV-Cre vectors had a relatively mild phenotype, with reduced body weight gain probably as a result of reduced food intake.

We previously showed that short-term AAV-shRNA knockdown of *Fto* in the hypothalamic ARC of rats led to increased food intake, although there was no effect on body weight [33]. These apparent differences may, in part, be due to different hypothalamic areas being targeted; in the present mouse-based study, the mediobasal hypothalamus was targeted, whereas the arcuate nucleus alone was targeted in the previous rat study [33]. In contrast, to our AAV-Cre knockout a neural specific knockout of *Fto* has been reported that used a nestin Cre to delete a floxed Fto exon 3. These mice have an overt phenotype very similar to constitutive global KO mice [27]. Nestin is expressed far more widely than the hypothalamus, and is expressed early in development (from embryonic day 7.75 [37]), which may explain the differences with our AAV-Cre knockout. Global deletion of *Fto* in adult onset KO mice will target multiple brain regions as *Fto* is very widely expressed [38].

FTO is expressed throughout the body and is particularly highly expressed in many regions within the brain [1], [38]. In keeping with a gene implicated as having a potential role in appetitive behaviour, the hypothalamic expression of *Fto* in response to a variety of nutritional changes has been extensively studied [38]–[42]. A clear, consistent message from these studies is difficult to ascertain with *Fto* expression having been reported to be down regulated [1], [39]–[40], unchanged [38] and also up-regulated 41–42 in response to fasting. These differences are likely to be a reflection of a range of fasting duration, strain and species differences as well as variations in tissue studied (whole hypothalamus vs individual regions of hypothalamus). However, it may be that some of variability in these data is driven by a particular facet of metabolic state of the different animals during the study period. For example, we have recently reported that essential amino acid starvation reduces FTO protein and mRNA levels in cell lines and that this is reversible on re-feeding [36].

Male and female mice differ in body weight and body composition and female mice also go through an estrous cycle of 4–6 days. The other published global germline knockouts show clear and similar effects in both male and female mice, although the differences in females are not as overt as in male and in the case of the Gao *et al.* knockout (2010) there is as already noted gradual convergence in female body weights over 3 to 12 weeks. Similarly for our knockout female mice are not as strongly affected as males (data not shown). These differences are unlikely to reflect basic differences in the mechanism of FTO action but more likely in the relative ability of the sexes to buffer changes in metabolism and in sex hormone dependent difference in growth and behaviour.

The effect of adult onset global deletion of *Fto* on lean mass suggests that it could be interesting to examine *FTO* genotype in conditions of cachexia to see if there is a modifying effect. An initial study in chronic obstructive pulmonary disease (COPD) suggests variation of lung function with FTO genotype and proposes a link with cachexia in a subset of these patients [43].

Our data indicate that FTO has a clear role in influencing body composition and metabolic substrate utilisation within peripheral tissues. They also show that total removal of FTO can be as deleterious as increasing its expression, as both adult onset KO mice and mice globally overexpressing *Fto* mice have increased fat mass [32]. This suggests that in man the at-risk allele could influence BMI either by decreasing or increasing FTO levels, or by impairment of its proper regulation. There are possible parallels between the early adiposity rebound observed in children with the at-risk A allele and adult onset KO mice which show an initial decrease in body weight before later increasing weight [10]–[11].

Whether FTO is a good drug target for obesity is still an open question despite there being some undesirable effects of knocking it out. There may be a therapeutic range of inhibition that would be beneficial. Formally it also remains to be proven that the catalytic activity is solely responsible for the effects that have been shown by gene manipulation.

In conclusion, our data demonstrate that the FTO phenotype is not linked to energy expenditure, that FTO has a role in early life and that germline loss leads to significant lethality, that loss in adulthood is better tolerated and leads to changes in both lean and fat mass (through alterations in metabolism) and that hypothalamic loss of *Fto* only explains a small part of the phenotype. Thus our study shows a complex role for FTO both in the brain, in controlling food intake, and in peripheral metabolism and substrate utilisation.

## Materials and Methods

### Animal Husbandry

All animal studies were carried out in accordance with UK Home Office legislation and local ethical guidelines issued by the Medical Research Council (Responsibility in the Use of Animals for Medical Research, July 1993). Mice were kept under controlled light (light 7am–7pm, dark 7pm–7am), temperature (21±2°C) and humidity (55±10%) conditions. They had free access to water (9–13 ppm chlorine). They were fed *ad libitum* on a commercial diet (SDS Rat and Mouse No. 3 Breeding diet, RM3) containing 11.5 kcal% fat, 23.93 kcal% protein and 61.57 kcal% carbohydrate. Actin-cre and tamoxifen inducible ubiquitin-Cre mice were obtained from the Jackson Laboratory (Stock name Tg(ACTA1-cre)79Jme/J and B6.Cg-Tg(UBC-cre/ERT2)1Ejb/J, respectively). Phenotyping tests were performed according to EMPReSS (European Phenotyping Resource for Standardised Screens from EUMORPHIA) standardized protocols as described at (http://empress.har.mrc.ac.uk).

### Construction of *Fto* Targeting Vector

The *Fto* exon 3 conditional knockout construct was designed to introduce LoxP sites flanking exon 3 and a FRT flanked neomycin resistance cassette. A MC1 driven thymidine kinase cassette (PL253; http://recombineering.ncifcrf.gov/) was subcloned into the XhoI site of PL451 (http://recombineering.ncifcrf.gov/) to generate pTKNEO. Additional restriction sites (PmlI) were added to PL451 using asymmetric overlapping oligonucleotides (Table S7) into the NotI site of PL451 to generate p3OLI. An additional LoxP site was inserted by asymmetric overlapping oligonucleotides into the ClaI site of pTKNEO (Table S7). A *Fto* intron-two 5′ recombinogenic arm of homology was amplified (3496 bp) from R1 129 ES cell genomic DNA using Phusion High-Fidelity PCR (Finnzymes, NEB) using the oligonucleotides 5′-CATTCTTTTATTTTGCCTGAGTGTG-3′ and 5′- ATACAAAACCAACGCCCAGACA-3′ and subcloned into the PmeI site of PTKNEO to generate pTKNEO5′. Next *Fto* exon 3 was amplified (830 bp) using the oligonucleotides 5′-GCTGGGGAAAAGTACTGTGTAGTTT-3′ and 5′-ACAGACACGAGTGTGCTTACTCATC-3′ and subcloned into the HpaI site of pTKNEO5′ to generate pTKNEO5′Ex3. An *Fto* intron three 3′ recombinogenic arm of homology was amplified (3437 bp) using the oligonucleotides 5′-TATATCTGCAGAGCACCCCTCTCC-3′ and 5′-ACAATCCAACAATAGCAGGAGCA-3′ and subcloned into the PmeI site of p3OLI to generate p3OLI3arm. Finally this plasmid was digested with NotI and the 3′ recombinogenic arm of homology was cloned into the NotI site of pTKNeo5′Ex3 to generate the *Fto* Exon 3 targeting vector.

### Gene Targeting

R1 129 embryonic stem (ES) cells were transfected with 20 µg of SrfI linearized vector. ES cell clones were selected by ganciclovir and G418 (Geneticin) and screened by Southern blot analysis using approximately 10 µg of *Hind*III digested genomic DNA. A 5′ probe was generated by amplification of 129 R1 genomic DNA with 5′- GGCATGTTAGCTGTCTCAGCCT-3′ and 5′-CTGTTGACACTGCTGATTCCTG-3′. A 3′ probe was generated by amplification of 129 R1 genomic DNA with 5′- CTGTTACCATGTTGTTGGGTTT-3′ and 5′-CAGAGCTAATGATACTCACTTCGC-3′. Radiolabelled probes were produced using a Rediprime II DNA labelleling system (GE Healthcare Life Sciences).

Successfully targeted ES cell clones were injected into C57BL/6J blastocysts (Transgenesis and Gene Targeting, MRC Harwell). The resulting chimeras were bred to C57BL/6J mice and offspring (F1 individuals) tested for germline transmission. Mice were genotyped by PCR using Phusion High-Fidelity PCR (Finnzymes, NEB) with 5′- AGCGCTCACTGGAGAGTGTCTG-3′ and 5′- GAGCCAGAGAGGATTTAGATGGG-3′ producing wildtype (989 bp), floxed (1184 bp) or Cre recombined knockout (237 bp) amplification products.

#### Global constitutive KO allele

F1 mice were crossed to β-actin-Cre recombinase mice (Jackson Laboratory: Stock name Tg(ACTA1-Cre)79Jme/J) congenic to C57BL/6J and the progeny genotyped for Cre recombinase. Subsequent mice were backcrossed to C57BL/6J to remove Cre recombinase.

#### Conditional floxed allele stock

Independent F1 mice were also crossed to a line carrying the improved thermostable Flp(e) recombinase under the control of the β-actin promoter [44], congenic to C57BL/6J, to remove the *Neo* selection cassette. Following Flpe-mediated excision of *Neo*, Flp(e) recombinase was segregated by crossing to C57BL/6J mice.

#### Generation of the global adult knockout and control mice

The *Fto* conditional floxed allele was crossed with a tamoxifen inducible Cre line from the Jackson laboratories (B6.Cg-Tg(UBC-Cre/ESR1)1Ejb/J). At 6 weeks of age male mice were treated with either 200 mg/kg tamoxifen free base solution (MP Biomedicals, Santa Ana, CA) dissolved in corn oil 2% ethanol solution, or an equivalent amount of vehicle by oral gavage for 5 consecutive days.

### Body Mass and Composition

Body mass was measured each week on scales calibrated to 0.01 g. Analysis of body composition was performed by DEXA using the Lunar PIXImus Mouse Densitometer (Wipro GE Healthcare, Madison, WI) or with an Echo MRI whole body composition analyzer (Echo Medical System, Houston, TX).

### Measurement of Food Consumption

Food consumption in germline loss and adult onset loss groups was measured when mice were housed in metabolic Techniplast cages. Food consumption in AAV-Cre treated mice was measured while mice were housed in home cage.

### Metabolic Rate Measurements

Metabolic rate was measured at 18 weeks of age using indirect calorimetry (Oxymax; Columbus Instruments) to determine oxygen consumption, carbon dioxide production, respiratory exchange ratio (RER) and heat production. Heat production (energy expenditure) was calculated using; Heat = CV×VO_2_, CV = 3.815+1.232×RER (CV, calorific value based on the observed respiratory exchange ratio; Oxymax; Columbus Instruments).

### Intraperitoneal Glucose Tolerance Test

Mice were fasted overnight (16 hours) to establish a baseline glucose level “T0” (time zero). Mice were weighed, and a blood sample collected from the tail vein after administration of local anaesthetic (EMLA cream, Eutectic Mixture of Local Anaesthetics, Lidocaine/Prilocaine, AstraZeneca, UK) using Lithium-Heparin microvette tubes (Sarstedt, Germany). They were then injected intraperitoneally with 2 g glucose/kg body weight (20% glucose in 0.9% NaCl). Blood samples were taken at 60 and 120 min (or 10, 20 and 30 min) after injection. Plasma glucose was measured using an Analox Glucose Analyser GM9 (Analox, UK). Plasma insulin was measured using a Mercodia ultrasensitive mouse ELISA kit (Mercodia, Sweden). AUC analysis was performed using GraphPad Prism version 5.02 for Windows.

### Stereotactic Surgery

Nine to eleven week old homozygous *Fto*-floxed females were stereotactically injected with AAV vectors while under anesthesia. The coordinates used for the injections were determined using Paxinos et al. [45] and were 1.6 mm caudal to bregma, ±0.25 mm lateral to the midline and 6.0 mm below the surface of the skull in all cases. Using a 5 µl Hamilton syringe, 200 nl AAV vectors were injected into each side of the hypothalamus over a 1 min period. After delivery of the AAV vectors the needle was left in place for 10 min to prevent reflux. Body weight measurements were recorded every week for 8 weeks. Daily food intake was recorded between days 14 and 24 post injection.

### Immunohistochemistry

Eight weeks post injection, animals were anesthetized with Dolethal then transcardially perfused with 20 ml PBS followed by 40 ml 10% Formalin (Sigma). Brains were dissected out and incubated in 15% sucrose/10% formalin overnight at 4°C. Following cryoprotection in 30% sucrose/PBS, brains were frozen on dry-ice and stored at −80°C overnight. Serial 35 µM sections were taken using a freezing microtome and mounted on glass slides (VWR). Antibodies used were: FTO (custom rabbit anti recombinant mFTO antibody, 1∶3000 dilution in 5% goat serum/0.3% Triton X-100) and Cre (Novagen, 1∶1000 dilution in goat serum/0.3% Triton X-100). Biotinylated goat-anti-rabbit IgG (Vector Laboratories, 1∶300 dilution) was used as a secondary antibody. FTO protein was visualized using avidin-Texas Red (Vector Labs, 1∶50 dilution in PBS) and sections were counterstained using DAPI. Cre protein was visualized using the Vectastain ABC elite kit and VIP peroxidase kit (Vector Labs).

### Western Blot Analysis and Antibodies

Western blots were performed on 50 ug of total proteins using a custom made rabbit anti recombinant mFTO antibody. Detection was performed using ultrasensitive horseradish Enhanced Chemiluminescence Plus (ECL plus; Amersham).

### Statistics

Error bars show the standard error (SE). Unless otherwise stated, p-values correspond to a Welch *t*-test of the null hypothesis of no difference between genotypic groups. R (http://www.r-project.org/) was used for the statistical analysis and presentation of results.

#### Correction for body size

When comparing some phenotypes across (treatment-genotype) groups, adjustment was made for variation in lean mass using multiple linear regression analysis (ANCOVA). Phenotypes for which this adjustment was made were: VO_2_ consumed, VCO_2_ produced, and energy expenditure (EE). The linear model used was:
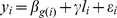
where

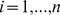
 indexes mouse,

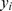
 denotes the phenotype of mouse 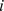
_,_

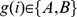
 denotes the (treatment-genotype) group of mouse 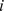,
- the 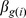 denote the main group effects

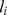
 is the lean mass of mouse 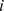, where 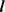 has been centred to have zero mean (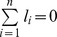)

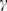
 is the coefficient for lean mass in the linear model
- the 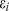 are mutually independent identically distributed zero-mean Gaussian random measurement errors.

Table S3 (indirect calorimetry analysis) and Figure 2, Figure 5 and Figure 7 (indirect calorimetry scatter plot data) show estimates for 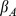, 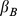, and 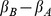, as well as a *p*-value for the test of the hypothesis 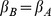. For the corrected phenotypes, the points plotted in Figure 2, Figure 5 and Figure 7 (indirect calorimetry scatter plot data) are lean mass corrected using the least-squares estimate of 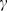 (i.e. the points 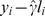 are plotted).

This approach was also applied to lean and fat mass adjustment for body weight (Table S2).

#### Repeated-measures ANOVA

Time-course data were analysed using the repeated-measures ANOVA model [46]:
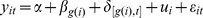
where

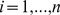
 indexes mouse,

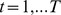
 indexes time point,

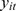
 denotes the phenotype of mouse 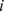 at time point 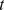,

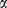
 denotes an intercept term

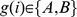
 denotes the (treatment-genotype) group of mouse 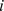,
- the 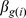 denote the main group effects (with the constraint 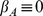)
- the 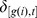 denote interactions between group and time (with the constraints 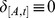 and 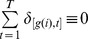),

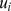
 is the random effect for mouse 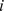 (these random effects model the correlation between repeated measures), with the 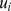 mutually independent identically distributed zero-mean Gaussian random variables, and
- the 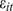 are mutually independent identically distributed zero-mean Gaussian random measurement errors.

The model was fitted using the lmer() function in the lme4 R package [47]. The null hypothesis of no main genotypic effect (i.e. that 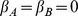 for both groups) was tested against the alternative hypothesis under which 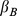 was unconstrained. The test was based on the asymptotic 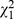 null distribution of 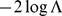, where 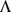 denotes the likelihood ratio. To supplement the repeated-measures analysis, Welch t-tests were performed separately at each time point (Tables S1, S4, S5, S6 and S8).

## Supporting Information

Figure S1 Recombinase Mediated Excision of *Neo* and *Fto* Exon 3. A. Schematic of the generation of WT, conditional knock-out (*Neo* removed by Flp recombination; *Fto* ^+/*Flox*^) and knockout (exon 3 deletion; *Fto* ^+/−^) mice. Southern blot analysis of targeted ES clones. Genomic DNA digested with *Hind*III and probed with a 5′external *Fto* probe to confirm targeting (Tg) of the *Fto* locus. WT produces a 12.5 kb band whereas the targeted locus generates a 7.2 kb band. B. Representative immunoblot of FTO (56 kDa) and actin (loading control, 42 kDa) in adult brain, liver and 17.5 dpc embryo body, from *Fto* ^−/−^, *Fto* ^+/−^ and WT mice.
(DOCX)

Figure S2 Loss of FTO in male global adult onset KO mice. Immunoblot demonstrating loss of FTO protein in whole brain, epigonadal white adipose tissue (Epi WAT) and liver of adult onset KO mice but not in control mice (tamoxifen-treated Cre and vehicle-treated). Tissue collected at 20 weeks of age (14 weeks post treatment).
(DOCX)

Figure S3 FTO expression in female Adult hypothalamic KO mice. A & B Consecutive sections through the hypothalamus of a mouse unilaterally injected with AAV-Cre vectors showing Cre recombinase expression (A, purple staining) and FTO expression (B, red staining). FTO expression is absent in the region of the brain where Cre expression is found. C–E Representative images showing normal FTO protein expression (red staining) within the hypothalamus of a sham injected mice 8 weeks after surgery. F–H Representative images showing dramatically reduced Fto protein expression 8 weeks after injection of AAV-Cre vectors into the mediobasal hypothalamus.
(DOCX)

Figure S4 Hypothalamic protein expression 8 weeks post surgery in female adult onset hypothalamic KO mice. A Representative image showing a western blot to detect expression of Fto, Beta Tubulin, GFP or Cre in the brains of female homozygous floxed mice injected with either an AAV-GFP (Sham) or AAV-Cre. 8 weeks following injection, mice were sacrificed, their hypothalamus and cortex sub-dissected and proteins extracted using RIPA buffer. GFP expression was only detected in the hypothalmi of mice injected with an AAV-GFP (Sham) and Cre expression was only detected in the hypothalmi of AAV-Cre injected mice. B Image J software was used to quantify the intensity of Fto and Beta tubulin protein bands from 2 animals. When normalised for Beta Tubulin expression, an approximate 50% decrease in Fto expression was seen in the hypothalami, but not cortex, of AAV-Cre injected mice.
(DOCX)

Figure S5 Energy expenditure in female adult onset hypothalamic KO mice. A, VO2 consumed, B, CO2 produced. Data are expressed as mean ± SE. Female adult AAV-Cre treated (n = 5) and sham control (n = 5) mice. Measurements were made, 5 weeks after treatment, over a 48-hour period during the dark (lights out) and light (lights on) phases.
(DOCX)

Table S1 Time by time ANOVA analysis of weight in global germline *Fto* KO mice. s.e, standard error.
(DOCX)

Table S2 Analysis of fat and lean mass data either as raw data, data normalised by multiple linear regression (ANCOVA) for body weight or by % of body weight. GG, Global Germline Knockout; GAO, Global Adult Onset knockout; AAV, hypothalamic adult onset knockout using AAV Cre, s.e, standard error.
(DOCX)

Table S3 Energy expenditure phenotypes across (treatment-genotype) groups, with the exception of RER, adjustment was made for variation in lean mass using multiple linear regression (ANCOVA). GG, Global Germline Knockout; GAO, Global Adult Onset knockout; AAV, hypothalamic adult onset knockout using AAV Cre, Light or dark phase; day or night, Tam; Tamoxifen, s.e; standard error.
(DOCX)

Table S4 Time by time ANOVA analysis of weight in global adult onset mice. s.e, standard error.
(DOCX)

Table S5 Time by time ANOVA analysis of weight in hypothalamic adult onset mice. s.e, standard error.
(DOCX)

Table S6 Time by time ANOVA analysis of baseline standardised weight in hypothalamic adult onset mice. s.e, standard error.
(DOCX)

Table S7 Oligonucleotides for introducing 5′ LoxP site (LoxPT, LOXPB) and oligonucleotides for introducing additional restriction sites into PL451 (FTO3CST, FTO3CSB).
(DOCX)

Table S8 Repeated measures ANOVA analysis of body weight. GG, Global Germline Knockout; GAO, Global Adult Onset knockout; AAV, hypothalamic adult onset knockout using AAV Cre, s.e, standard error.
(DOCX)
