# Supplementary figures and images for: Adult Onset Global Loss of the Fto Gene Alters Body Composition and Metabolism in the Mouse
Source: PLoS Genet. 2013 Jan 3;9(1):e1003166. doi: 10.1371/journal.pgen.1003166 (PMC3536712; doi:10.1371/journal.pgen.1003166)

**
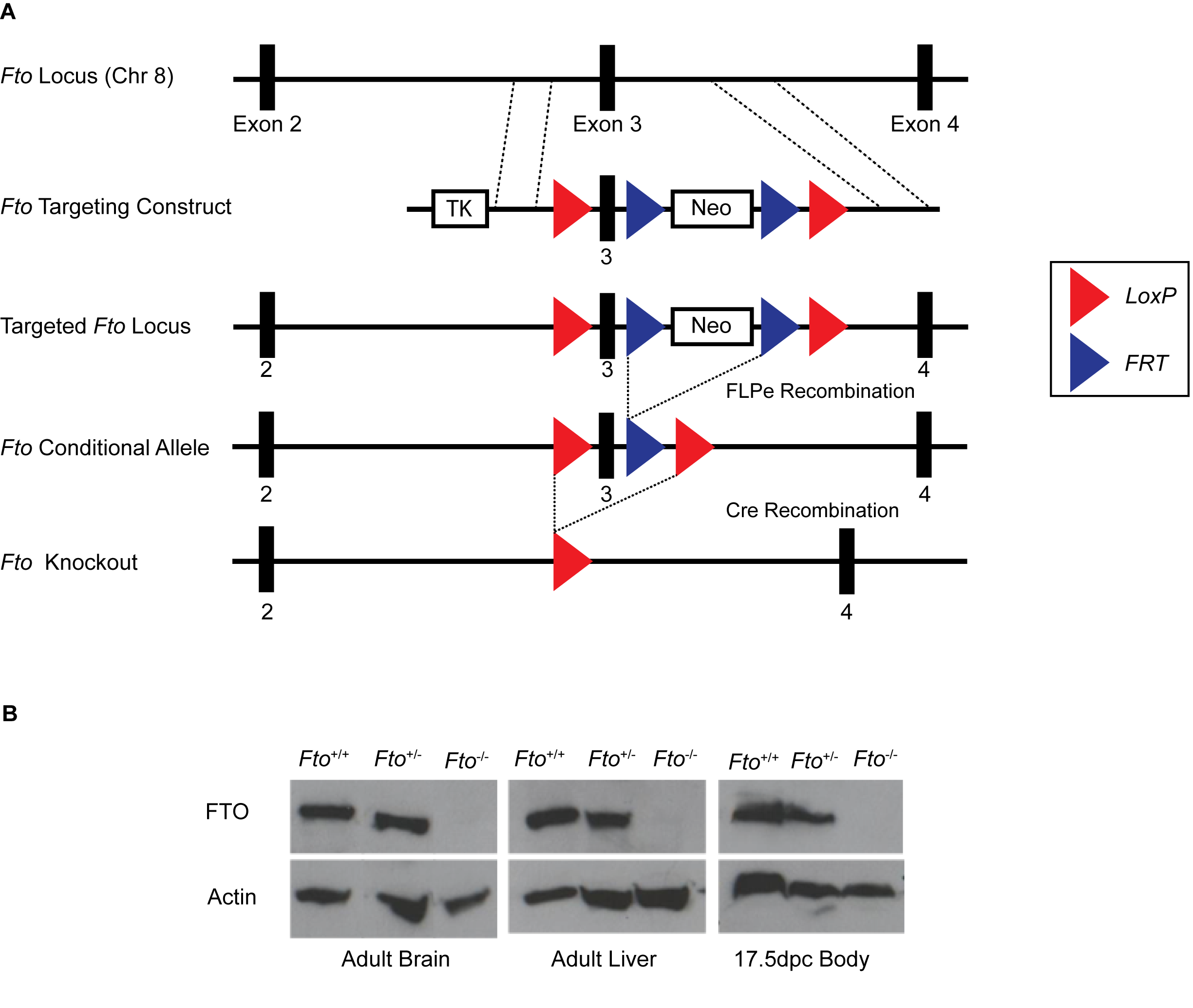
**

Supplement: Figure S1 — Recombinase Mediated Excision of Neo and Fto Exon 3. A. Schematic of the generation of WT, conditional knock-out (Neo removed by Flp recombination; Fto +/Flox) and knockout (exon 3 deletion; Fto +/−) mice. Southern blot analysis of targeted ES clones. Genomic DNA digested with HindIII and probed with a 5′external Fto probe to confirm targeting (Tg) of the Fto locus. WT produces a 12.5 kb band whereas the targeted locus generates a 7.2 kb band. B. Representative immunoblot of FTO (56 kDa) and actin (loading control, 42 kDa) in adult brain, liver and 17.5 dpc embryo body, from Fto −/−, Fto +/− and WT mice. (DOCX) [file pgen.1003166.s001.docx]

**
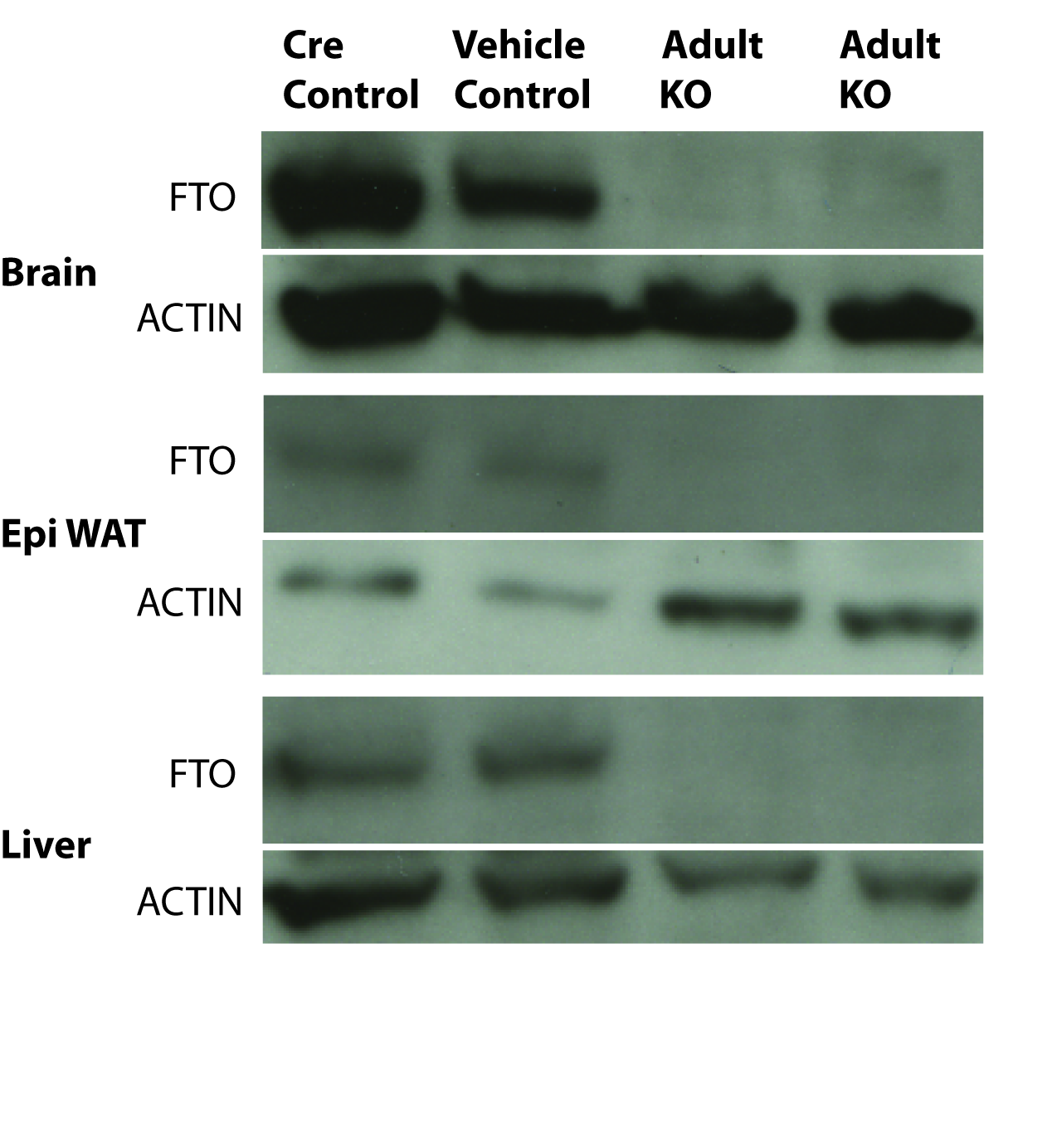
**

Supplement: Figure S2 — Loss of FTO in male global adult onset KO mice. Immunoblot demonstrating loss of FTO protein in whole brain, epigonadal white adipose tissue (Epi WAT) and liver of adult onset KO mice but not in control mice (tamoxifen-treated Cre and vehicle-treated). Tissue collected at 20 weeks of age (14 weeks post treatment). (DOCX) [file pgen.1003166.s002.docx]

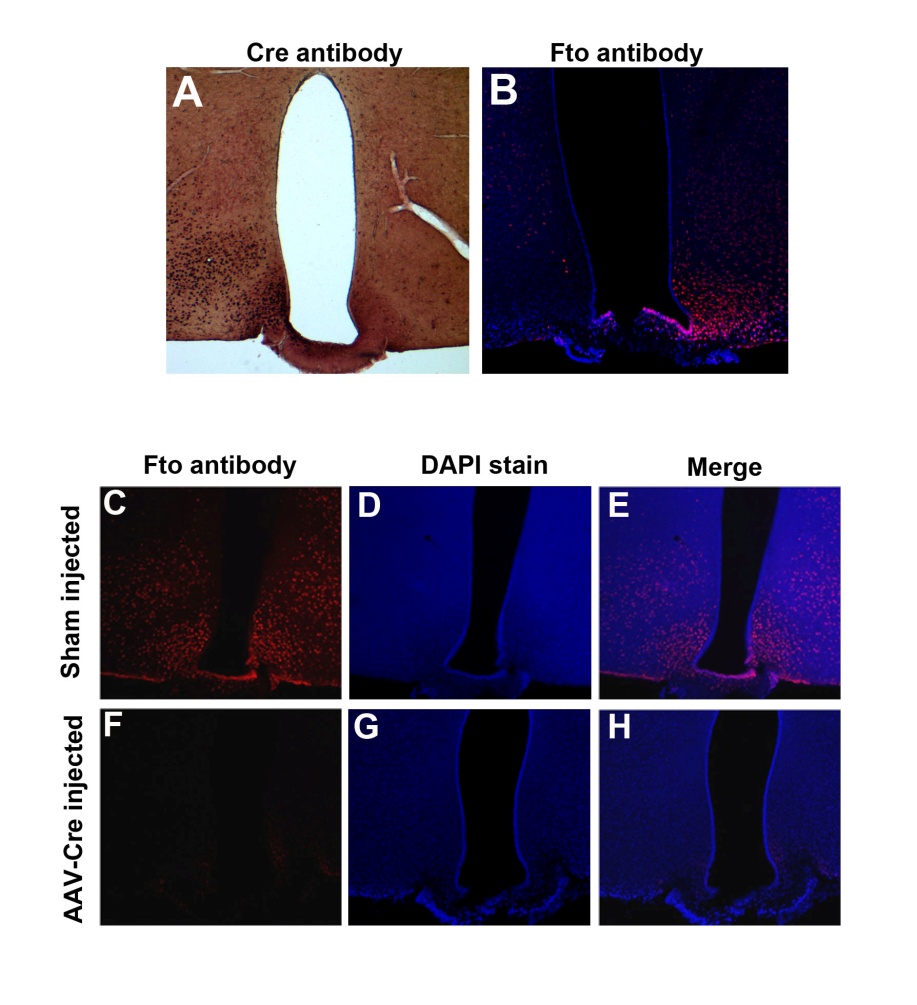

Supplement: Figure S3 — FTO expression in female Adult hypothalamic KO mice. A & B Consecutive sections through the hypothalamus of a mouse unilaterally injected with AAV-Cre vectors showing Cre recombinase expression (A, purple staining) and FTO expression (B, red staining). FTO expression is absent in the region of the brain where Cre expression is found. C–E Representative images showing normal FTO protein expression (red staining) within the hypothalamus of a sham injected mice 8 weeks after surgery. F–H Representative images showing dramatically reduced Fto protein expression 8 weeks after injection of AAV-Cre vectors into the mediobasal hypothalamus. (DOCX) [file pgen.1003166.s003.docx]

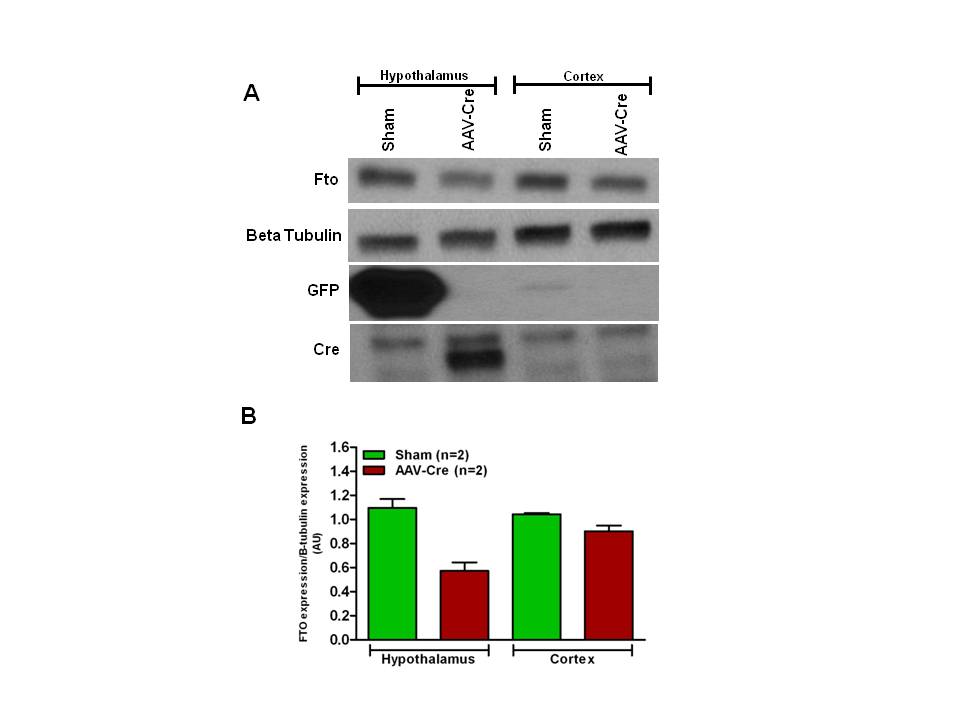

Supplement: Figure S4 — Hypothalamic protein expression 8 weeks post surgery in female adult onset hypothalamic KO mice. A Representative image showing a western blot to detect expression of Fto, Beta Tubulin, GFP or Cre in the brains of female homozygous floxed mice injected with either an AAV-GFP (Sham) or AAV-Cre. 8 weeks following injection, mice were sacrificed, their hypothalamus and cortex sub-dissected and proteins extracted using RIPA buffer. GFP expression was only detected in the hypothalmi of mice injected with an AAV-GFP (Sham) and Cre expression was only detected in the hypothalmi of AAV-Cre injected mice. B Image J software was used to quantify the intensity of Fto and Beta tubulin protein bands from 2 animals. When normalised for Beta Tubulin expression, an approximate 50% decrease in Fto expression was seen in the hypothalami, but not cortex, of AAV-Cre injected mice. (DOCX) [file pgen.1003166.s004.docx]
