# Supplementary material for: Adult Onset Global Loss of the Fto Gene Alters Body Composition and Metabolism in the Mouse
Source: PLoS Genet. 2013 Jan 3;9(1):e1003166. doi: 10.1371/journal.pgen.1003166 (PMC3536712; doi:10.1371/journal.pgen.1003166)
Supplement: Table S1 — Time by time ANOVA analysis of weight in global germline Fto KO mice. s.e, standard error. (DOCX) [file pgen.1003166.s006.docx]

| **Week** | **Wildtype mean (s.e.)** | **KO mean (s.e.)** | **p value** |
| --- | --- | --- | --- |
| 4 | 18.8 (0.6) | 12.7 (0.6) | 6.40E-07 |
| 5 | 22.0 (0.5) | 17.8 (0.7) | 9.50E-05 |
| 6 | 24.0 (0.4) | 19.3 (0.5) | 2.70E-06 |
| 7 | 25.5 (0.5) | 20.6 (0.6) | 4.00E-06 |
| 8 | 26.3 (0.5) | 21.4 (0.5) | 1.30E-06 |
| 9 | 26.9 (0.5) | 21.6 (0.5) | 9.00E-07 |
| 10 | 28.4 (0.6) | 22.3 (0.5) | 5.10E-07 |
| 11 | 28.2 (0.6) | 22.8 (0.6) | 3.30E-06 |
| 12 | 28.7 (0.5) | 23.4 (0.5) | 1.70E-06 |
| 13 | 27.9 (0.6) | 23.8 (0.6) | 0.00014 |
| 14 | 28.8 (0.7) | 24.1 (0.5) | 4.40E-05 |
| 15 | 28.1 (1.0) | 25.2 (0.5) | 0.024 |
| 16 | 29.6 (0.8) | 25.4 (0.5) | 0.00047 |
| 17 | 30.0 (0.9) | 26.3 (0.6) | 0.0029 |
| 18 | 31.8 (0.5) | 26.7 (0.9) | 0.00015 |
| 19 | 31.8 (0.6) | 27.5 (0.8) | 0.00067 |
| 20 | 31.1 (0.6) | 27.0 (0.9) | 0.0016 |
