# Supplementary material for: Adult Onset Global Loss of the Fto Gene Alters Body Composition and Metabolism in the Mouse
Source: PLoS Genet. 2013 Jan 3;9(1):e1003166. doi: 10.1371/journal.pgen.1003166 (PMC3536712; doi:10.1371/journal.pgen.1003166)
Supplement: Table S2 — Analysis of fat and lean mass data either as raw data, data normalised by multiple linear regression (ANCOVA) for body weight or by % of body weight. GG, Global Germline Knockout; GAO, Global Adult Onset knockout; AAV, hypothalamic adult onset knockout using AAV Cre, s.e, standard error. (DOCX) [file pgen.1003166.s007.docx]

| **Study** | **Weeks** | **Trait**  **(g)** | **Method** | **Estimate** | **s.e.** | **t value** | **Pr(>\|t\|)** |
| --- | --- | --- | --- | --- | --- | --- | --- |
| GG | 20 | fat | Raw | -1.875 | 0.71 | -2.64 | 0.015 |
| GG | 20 | fat | ANCOVA | -1 | 1.992 | -0.502 | 0.62 |
| GG | 20 | fat | % of BW | -0.029 | 0.022 | -1.285 | 0.21 |
| GG | 20 | lean | Raw | -7.512 | 0.671 | -11.191 | 8.80E-11 |
| GG | 20 | lean | ANCOVA | -4.379 | 1.752 | -2.5 | 0.02 |
| GG | 20 | lean | % of BW | -0.115 | 0.022 | -5.254 | 2.50E-05 |
|  |  |  |  |  |  |  |  |
| GAO | 9 | fat | Raw | 0.491 | 0.237 | 2.072 | 0.05 |
| GAO | 9 | fat | ANCOVA | 0.682 | 0.28 | 2.433 | 0.024 |
| GAO | 9 | fat | % of BW | 0.028 | 0.009 | 3.142 | 0.0047 |
| GAO | 9 | lean | Raw | -2.805 | 0.622 | -4.513 | 0.00017 |
| GAO | 9 | lean | ANCOVA | -1.014 | 0.268 | -3.78 | 0.0011 |
| GAO | 9 | lean | % of BW | -0.041 | 0.008 | -4.783 | 8.90E-05 |
| GAO | 20 | fat | Raw | 3.318 | 0.442 | 7.508 | 3.10E-07 |
| GAO | 20 | fat | ANCOVA | 3.859 | 0.338 | 11.422 | 5.90E-10 |
| GAO | 20 | fat | % of BW | 0.115 | 0.011 | 10.344 | 1.80E-09 |
| GAO | 20 | lean | Raw | -4.206 | 0.554 | -7.588 | 2.60E-07 |
| GAO | 20 | lean | ANCOVA | -3.402 | 0.32 | -10.628 | 2.00E-09 |
| GAO | 20 | lean | % of BW | -0.102 | 0.01 | -9.949 | 3.40E-09 |
