# Supplementary material for: Adult Onset Global Loss of the Fto Gene Alters Body Composition and Metabolism in the Mouse
Source: PLoS Genet. 2013 Jan 3;9(1):e1003166. doi: 10.1371/journal.pgen.1003166 (PMC3536712; doi:10.1371/journal.pgen.1003166)
Supplement: Table S3 — Energy expenditure phenotypes across (treatment-genotype) groups, with the exception of RER, adjustment was made for variation in lean mass using multiple linear regression (ANCOVA). GG, Global Germline Knockout; GAO, Global Adult Onset knockout; AAV, hypothalamic adult onset knockout using AAV Cre, Light or dark phase; day or night, Tam; Tamoxifen, s.e; standard error. (DOCX) [file pgen.1003166.s008.docx]

| **Study** | **y** | **Time** | **A** | **A mean (s.e)** | **B** | **B mean (s.e)** | **B-A mean (s.e)** | **p-value** |
| --- | --- | --- | --- | --- | --- | --- | --- | --- |
| GG | VO2 | Light | WT | 84.7 (2.4) | KO | 97.2 (4.2) | 12.5 (5.7) | 0.037 |
| GG | VO2 | Dark | WT | 96.9 (2.6) | KO | 107.5 (4.6) | 10.6 (6.2) | 0.098 |
| GG | VCO2 | Light | WT | 74.9 (2.0) | KO | 82.0 (3.6) | 7.1 (4.9) | 0.16 |
| GG | VCO2 | Dark | WT | 91.0 (2.3) | KO | 93.3 (4.2) | 2.3 (5.6) | 0.69 |
| GG | RER | Light | WT | 0.87 (0.01) | KO | 0.83 (0.01) | -0.04 (0.02) | 0.041 |
| GG | RER | Dark | WT | 0.92 (0.01) | KO | 0.87 (0.02) | -0.05 (0.02) | 0.01 |
| GG | EE | Light | WT | 1.83 (0.05) | KO | 1.95 (0.09) | 0.12 (0.12) | 0.32 |
| GG | EE | Dark | WT | 2.13 (0.06) | KO | 2.10 (0.10) | -0.03 (0.13) | 0.84 |
|  |  |  |  |  |  |  |  |  |
| GAO | VO2 | Light | Vehicle | 93.1 (3.8) | Tam | 96.0 (3.3) | 2.9 (6.3) | 0.65 |
| GAO | VO2 | Dark | Vehicle | 107.6 (4.1) | Tam | 111.9 (3.6) | 4.3 (6.9) | 0.54 |
| GAO | VCO2 | Light | Vehicle | 84.6 (3.0) | Tam | 72.8 (2.6) | -11.9 (5.0) | 0.027 |
| GAO | VCO2 | Dark | Vehicle | 99.9 (2.6) | Tam | 85.6 (2.2) | -14.3 (4.3) | 0.0035 |
| GAO | RER | Light | Vehicle | 0.88 (0.01) | Tam | 0.78 (0.01) | -0.10 (0.01) | 8.10E-07 |
| GAO | RER | Dark | Vehicle | 0.91 (0.01) | Tam | 0.78 (0.01) | -0.12 (0.02) | 3.10E-06 |
| GAO | EE | Light | Vehicle | 1.95 (0.07) | Tam | 1.90 (0.06) | -0.05 (0.12) | 0.7 |
| GAO | EE | Dark | Vehicle | 2.24 (0.08) | Tam | 2.23 (0.07) | -0.01 (0.13) | 0.96 |
|  |  |  |  |  |  |  |  |  |
| AAV | VO2 | Light | Sham | 90.4 (3.1) | Cre | 91.9 (3.1) | 1.5 (4.4) | 0.74 |
| AAV | VO2 | Dark | Sham | 77.6 (2.5) | Cre | 77.3 (2.5) | -0.2 (3.6) | 0.95 |
| AAV | VCO2 | Light | Sham | 87.2 (3.5) | Cre | 91.1 (3.5) | 3.9 (5.1) | 0.46 |
| AAV | VCO2 | Dark | Sham | 68.7 (2.3) | Cre | 71.0 (2.3) | 2.3 (3.3) | 0.51 |
| AAV | RER | Light | Sham | 0.96 (0.02) | Cre | 0.99 (0.02) | 0.02 (0.02) | 0.38 |
| AAV | RER | Dark | Sham | 0.90 (0.02) | Cre | 0.91 (0.02) | 0.02 (0.03) | 0.61 |
| AAV | EE | Light | Sham | 1.88 (0.06) | Cre | 1.93 (0.06) | 0.04 (0.10) | 0.65 |
| AAV | EE | Dark | Sham | 1.58 (0.05) | Cre | 1.59 (0.05) | 0.01 (0.07) | 0.91 |
