# Supplementary material for: Adult Onset Global Loss of the Fto Gene Alters Body Composition and Metabolism in the Mouse
Source: PLoS Genet. 2013 Jan 3;9(1):e1003166. doi: 10.1371/journal.pgen.1003166 (PMC3536712; doi:10.1371/journal.pgen.1003166)
Supplement: Table S4 — Time by time ANOVA analysis of weight in global adult onset mice. s.e, standard error. (DOCX) [file pgen.1003166.s009.docx]

| **Week** | **Vehicle mean (s.e.)** | **Tamoxifen mean (s.e.)** | **p value** |
| --- | --- | --- | --- |
| 3 | 14.3 (0.7) | 14.7 (0.7) | 0.67 |
| 4 | 19.9 (0.5) | 19.3 (0.5) | 0.45 |
| 5 | 22.3 (0.5) | 22.1 (0.4) | 0.73 |
| 6 | 23.4 (0.5) | 23.0 (0.5) | 0.64 |
| 7 | 25.0 (0.5) | 24.2 (0.5) | 0.22 |
| 8 | 26.2 (0.5) | 24.2 (0.5) | 0.0082 |
| 9 | 27.2 (0.4) | 24.3 (0.5) | 0.00043 |
| 10 | 28.4 (0.4) | 25.2 (0.5) | 0.00019 |
| 11 | 29.0 (0.5) | 26.2 (0.5) | 0.00041 |
| 12 | 29.5 (0.5) | 26.9 (0.5) | 0.0019 |
| 13 | 30.0 (0.5) | 27.8 (0.6) | 0.0098 |
| 14 | 30.3 (0.6) | 27.8 (0.6) | 0.0047 |
| 15 | 31.1 (0.6) | 28.5 (0.7) | 0.011 |
| 16 | 31.6 (0.6) | 28.7 (0.7) | 0.0063 |
| 17 | 32.0 (0.7) | 29.6 (0.4) | 0.011 |
| 18 | 31.9 (0.7) | 30.1 (0.4) | 0.029 |
| 19 | 32.2 (0.6) | 30.8 (0.4) | 0.066 |
| 20 | 32.6 (0.6) | 31.1 (0.5) | 0.062 |
