# Supplementary material for: Adult Onset Global Loss of the Fto Gene Alters Body Composition and Metabolism in the Mouse
Source: PLoS Genet. 2013 Jan 3;9(1):e1003166. doi: 10.1371/journal.pgen.1003166 (PMC3536712; doi:10.1371/journal.pgen.1003166)
Supplement: Table S5 — Time by time ANOVA analysis of weight in hypothalamic adult onset mice. s.e, standard error. (DOCX) [file pgen.1003166.s010.docx]

| **Week** | **Sham mean (s.e.)** | **Cre mean (s.e.)** | **p value** |
| --- | --- | --- | --- |
| 0 | 19.3 (0.4) | 20.5 (0.5) | 0.071 |
| 1 | 19.9 (0.3) | 20.8 (0.3) | 0.069 |
| 2 | 20.9 (0.3) | 21.5 (0.3) | 0.17 |
| 3 | 21.8 (0.4) | 22.1 (0.3) | 0.48 |
| 4 | 21.9 (0.3) | 22.5 (0.4) | 0.33 |
| 5 | 22.1 (0.3) | 22.5 (0.4) | 0.55 |
| 6 | 22.4 (0.2) | 22.7 (0.4) | 0.62 |
| 7 | 22.8 (0.3) | 22.9 (0.4) | 0.74 |
| 8 | 23.7 (0.4) | 23.5 (0.4) | 0.79 |
