# Supplementary material for: Adult Onset Global Loss of the Fto Gene Alters Body Composition and Metabolism in the Mouse
Source: PLoS Genet. 2013 Jan 3;9(1):e1003166. doi: 10.1371/journal.pgen.1003166 (PMC3536712; doi:10.1371/journal.pgen.1003166)
Supplement: Table S6 — Time by time ANOVA analysis of baseline standardised weight in hypothalamic adult onset mice. s.e, standard error. (DOCX) [file pgen.1003166.s011.docx]

| **Week** | **Sham mean (s.e.)** | **Cre mean (s.e.)** | **p value** |
| --- | --- | --- | --- |
| 1 | 0.6 (0.2) | 0.3 (0.2) | 0.21 |
| 2 | 1.6 (0.1) | 1.0 (0.3) | 0.11 |
| 3 | 2.5 (0.2) | 1.6 (0.2) | 0.012 |
| 4 | 2.6 (0.2) | 2.0 (0.4) | 0.15 |
| 5 | 2.8 (0.2) | 2.0 (0.4) | 0.069 |
| 6 | 3.1 (0.3) | 2.2 (0.4) | 0.088 |
| 7 | 3.5 (0.2) | 2.4 (0.4) | 0.049 |
| 8 | 4.4 (0.4) | 3.0 (0.4) | 0.023 |
