# Supplementary material for: Adult Onset Global Loss of the Fto Gene Alters Body Composition and Metabolism in the Mouse
Source: PLoS Genet. 2013 Jan 3;9(1):e1003166. doi: 10.1371/journal.pgen.1003166 (PMC3536712; doi:10.1371/journal.pgen.1003166)
Supplement: Table S7 — Oligonucleotides for introducing 5′ LoxP site (LoxPT, LOXPB) and oligonucleotides for introducing additional restriction sites into PL451 (FTO3CST, FTO3CSB). (DOCX) [file pgen.1003166.s012.docx]

| **Name** | **Sequence** |
| --- | --- |
| LOXPT: | 5‟CGATGGCCGGCCGGCGCGCCTTAATTAAGTTTAAACAAGGTTTGTTTTTTAGACTGGCAAGGTAAGAACCCTATAACTTCGTATAATGTATGCTATACGAAGTTATGTTAACAT-3‟ |
| LOXPB: | 5‟CGATGTTAACATAACTTCGTATAGCATACATTATACGAAGTTATAGGGTTCTTACCTTGCCAGTCTAAAAAACAAACCTTGTTTAAACTTAATTAAGGCGCGCCGGCCGGCCAT-3’ |
| FTO3CST | 5’GATCCGCGGCCGCCTAGGGTGTTGGGTGTCCTTTCCTTTCCTGGCCGGTCTCACCAGCACGTGGCCCGGGCGC-3’ |
| FTO3CSB | 5’GGCCGCGCCCGGGCCACGTGCTGGTGAGACCGGCCAGGAAAGGAAAGGACACCCAACACCCTAGGCGGCCGCG-3‟ |
