# Supplementary material for: Adult Onset Global Loss of the Fto Gene Alters Body Composition and Metabolism in the Mouse
Source: PLoS Genet. 2013 Jan 3;9(1):e1003166. doi: 10.1371/journal.pgen.1003166 (PMC3536712; doi:10.1371/journal.pgen.1003166)
Supplement: Table S8 — Repeated measures ANOVA analysis of body weight. GG, Global Germline Knockout; GAO, Global Adult Onset knockout; AAV, hypothalamic adult onset knockout using AAV Cre, s.e, standard error. (DOCX) [file pgen.1003166.s013.docx]

| **Study** | **Data subset** | **Weeks** | **Baseline subtracted?** | **A** | **B** | **B-A mean (s.e.)** | **p-value** |
| --- | --- | --- | --- | --- | --- | --- | --- |
| GG | All | 4-20 (post-natal) | No | WT | KO | -4.7 (0.6) | 2.8E-07 |
| GAO | Pre-treatment | 3-6 (post-natal) | No | Vehicle | Tam | -0.2 (0.6) | 0.75 |
| GAO | Post-treatment | 7-20 (post-natal) | No | Vehicle | Tam | -2.2 (0.7) | 0.0043 |
| GAO | Post-treatment | 7-20 (post-natal) | Yes, week 6 | Vehicle | Tam | -2.0 (0.4) | 0.00011 |
| AAV | Post-treatment | 1-8 (post-treatment) | No | Sham | Cre | 0.4 (0.4) | 0.29 |
| AAV | Post-treatment | 1-8 (post-treatment) | Yes, week 0 | Sham | Cre | -0.8 (0.3) | 0.021 |
