# Supplementary material for: Adult Onset Global Loss of the Fto Gene Alters Body Composition and Metabolism in the Mouse
Source: PLoS Genet. 2013 Jan 3;9(1):e1003166. doi: 10.1371/journal.pgen.1003166 (PMC3536712; doi:10.1371/journal.pgen.1003166)
Supplement: Supplementary file 10 [file pgen.1003166.s005.docx]

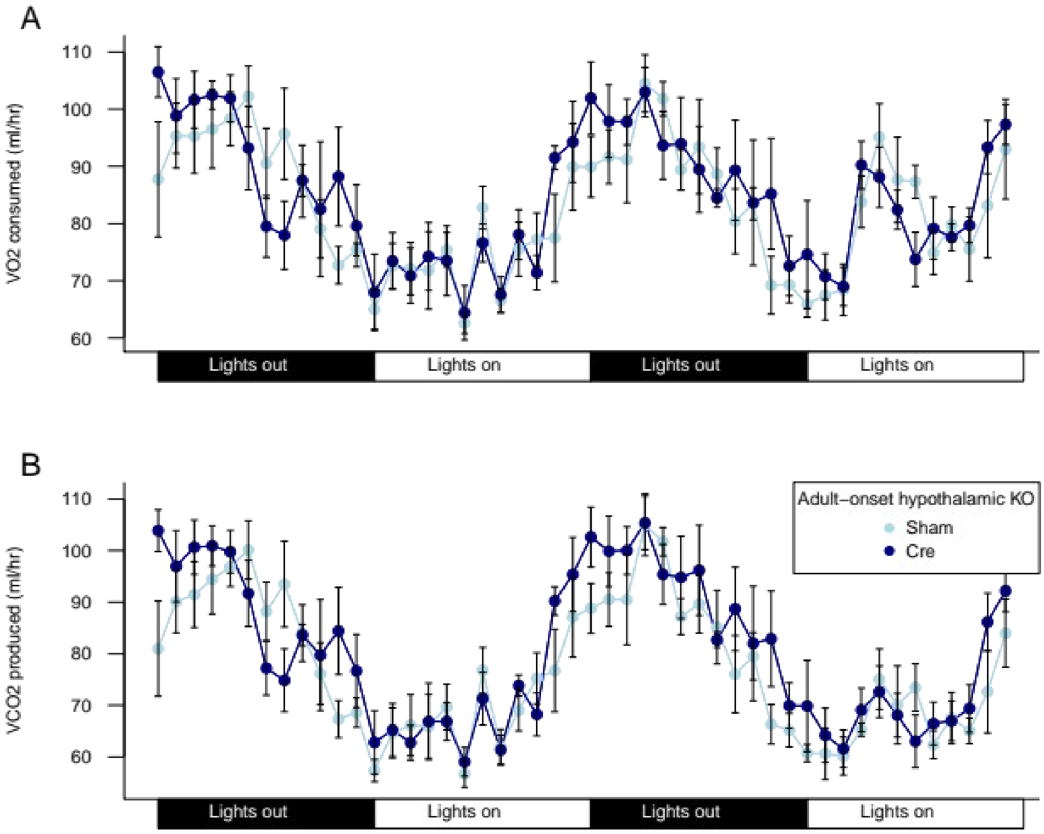
A
110
100
90
80
70
60
VO2 consumed (ml/hr)
Lights out
Lights on
Lights out
Lights on
B
Adult−onset hypothalamic KO
Sham
Cre
110
100
90
80
70
60
VCO2 produced (ml/hr)
Lights out
Lights on
Lights out
Lights on
